# Complement Components Showed a Time-Dependent Local Expression Pattern in Constant and Acute White Light-Induced Photoreceptor Damage

**DOI:** 10.3389/fnmol.2017.00197

**Published:** 2017-06-20

**Authors:** Nicole Schäfer, Antje Grosche, Sabrina I. Schmitt, Barbara M. Braunger, Diana Pauly

**Affiliations:** ^1^Department of Ophthalmology, University Hospital RegensburgRegensburg, Germany; ^2^Institute of Human Genetics, University RegensburgRegensburg, Germany; ^3^Institute of Human Anatomy and Embryology, University RegensburgRegensburg, Germany

**Keywords:** light-induced photoreceptor degeneration, complement system, retina, RPE/choroid, C3

## Abstract

**Background:** Photoreceptor cell death due to extensive light exposure and induced oxidative-stress are associated with retinal degeneration. A correlated dysregulation of the complement system amplifies the damaging effects, but the local and time-dependent progression of this mechanism is not thoroughly understood.

**Methods:** Light-induced photoreceptor damage (LD) was induced in Balb/c mice with white light illumination either for 24 h with 1000 lux (constant model) or 0.5 h with 5000 lux (acute model). Complement protein and mRNA expression levels were compared at 1 and 3 days post-LD for C1s, complement factor B (CFB), mannose binding lectin A, mannose-binding protein-associated serine protease 1 (MASP-1), C3, C4, C9, and complement factor P in retina and RPE/choroid. Histological analyses visualized apoptosis, microglia/macrophage migration, gliosis and deposition of the complement activation marker C3d. Systemic anaphylatoxin serum concentrations were determined using an ELISA.

**Results:** Apoptosis, gliosis and microglia/macrophage migration into the outer nuclear layer showed similar patterns in both models. Local complement factor expression revealed an early upregulation of complement factor mRNA in the acute and constant light regimen at 1 day post-treatment for *c1s*, *cfb*, *masp-1*, *c3*, *c4* and *c9* in the RPE/choroid. However, intraretinal complement mRNA expression for *c1s*, *cfb*, *c3* and *c4* was increased at 1 day in the constant and at 3 days in the acute model. A corresponding regulation on protein level in the retina following both LD models was observed for C3, which was upregulated at 1 day and correlated with increased C3d staining in the ganglion cell layer and at the RPE. In the RPE/choroid C1s-complex protein detection was increased at 3 days after LD irrespectively of the light intensities used.

**Conclusion:** LD in mouse eyes is correlated with local complement activity. The time-dependent local progression of complement regulation on mRNA and protein levels were equivalent in the acute and constant LD model, except for the intraretinal, time-dependent mRNA expression. Knowing the relative time courses of local complement expression and cellular activity can help to elucidate novel therapeutic options in retinal degeneration indicating at which time point of disease complement has to be rebalanced.

## Introduction

Exposure to sunlight has been suggested years ago as a risk factor for the development and progression of retinal degeneration and particularly age-related macular degeneration (AMD) [reviewed in Sui et al. (2013)]. Extensive light exposure induces apoptosis-driven photoreceptor loss in humans and mice (Wenzel et al., 2005; Organisciak and Vaughan, 2010). This is dependent on light intensity, light quality and exposure time (Wenzel et al., 2005). Several mechanisms are correlated with light-induced photoreceptor degeneration (LD), including an altered retinoid metabolism, increased oxidative stress and immunoreactions (Maeda et al., 2009; Pujol-Lereis et al., 2016). The exact interplay of these cascaded events still needs to be deciphered. Time-dependent analysis of the transcriptome after LD in mice revealed a clustered gene expression which imply a strongly regulated temporal coordination of stress gene expression in the light-damaged retina (Choi et al., 2001). However, we still lack an accurate understanding of the functional relevance of this time-dependent gene expression following LD and data that reveal how the transcriptomics are translated into protein expression.

The aim of the present study was to identify a LD-dependent transcriptional and protein expression profile of components of the complement system. Complement involvement in LD had been shown in a wide spectrum of white light treatment models, including mice/rats which were illuminated with light intensities between 1000 and 10000 lux for 1 h–10 days and evaluated after a recovery time up to 7 days (Rohrer et al., 2007; Rutar et al., 2011; Hadziahmetovic et al., 2012; Song et al., 2012). Comparison and generalization of these studies is difficult. Especially, since we know, that different light intensities, low and bright light, induce different apoptotic pathways in the retina (Roca et al., 2004) and prolonged recovery times influence the number of apoptotic cells following LD (Zhang et al., 2005; Jiao et al., 2015). Therefore, we chose a short-term, acute (0.5 h) and an intermediate, constant (24 h) exposure time with high (5000 lux) and medium (1000 lux) white light intensities to compare the effects of illumination on complement factor expression after 1 and 3 days of treatment (Supplementary Figures S1A–C).

The complement system is a humoral part of the innate immune system which bridges the adaptive and innate immune response. Genetic polymorphisms in the complement cascade, complement depositions in drusen and drugs targeting factors of the complement system as potential AMD-therapeutics, supposed a connection between retinal degeneration, complement expression and light irradiation (Anderson et al., 2010; Weber et al., 2014; Volz and Pauly, 2015). The complement system is composed of over 40 interacting proteins which are successively involved in the reaction. The cascade is activated by three distinct pathways: the classical, lectin and alternative pathways. Activation of all three pathways results in the cleavage of complement component C3 to C3a and C3b, with the latter being required for the cleavage of C5 to C5a and C5b as well as the formation of the terminal complement complex. The complement system finally results in three main biological functions: tagging of cell waste and pathogens by opsonins (e.g., iC3b), attraction and activation of immune cells by anaphylatoxins (e.g., C3a, C5a) and cell lysis by the terminal complement complex (C5b-C9). The activity of the complement system is tightly controlled under physiological conditions, but it can be dysregulated in pathological events resulting in an autoreactive damage of cells.

Previous studies reported that *c1q/s*, *c2*, *c3* and *c4* mRNA expression was upregulated in the retina after LD (Lohr et al., 2006; Rohrer et al., 2007; Rutar et al., 2011; Hadziahmetovic et al., 2012; Song et al., 2012), which was associated with microglia/macrophage migration (Rutar et al., 2011) and complement activated cell lysis (Lohr et al., 2006). Complement reaction after light treatment had been also suggested as an important pathway as complement factor D deficient mice were protected from LD (Rohrer et al., 2007).

To obtain a conclusive picture, here we correlate the time-dependent glial activation pattern, neuronal cell loss and expression of different complement factors on mRNA and, importantly, on protein level in the retina and RPE/choroid. Our results confirm previously described expression patterns regarding *c1s*, *c3* and *c4* and we added analyses regarding *cfb*, *masp-1*, *mbl*-a, *c9* and *cfp*. We describe an early gene upregulation in the RPE/choroid after 1 day of LD in both tested light models and in the retina for the constant light regime. Complement gene expression showed a delayed increase in the retina at 3 days following acute LD. We report for the first time, that C3 and C1s were altered on protein level in Western blots following both constant and acute LD either in the retina or in the RPE/choroid, respectively. Overall the studies revealed that C3, C1s and C9 could serve as time-dependent marker proteins for complement involvement for LD in mice.

## Materials and Methods

### Animals, Ethics Statement, and LD

Six to nine week old BALB/c mice obtained from Charles River Laboratories (Wilmington, MA, United States) and tested homozygous for the L450 variant of RPE65 (Wenzel et al., 2001) were kept in cyclic light (12 h on/12 h off, lights on at 7 am, light intensity approx. 400 lux) (Supplementary Figures S1A–C). Mouse experiments were strictly performed according to the guidelines of replacement, refinement, and reduction of animals in research (Russell and Burch, 1959) and approved by the committee on the ethics of animal experiments of the regional agency for animal health *Regierung der Oberpfalz, Veterinärwesen* (54-2532.1-04/11). To induce LD in mice, they were transferred to cyclic dim light (<100 lux) for 5 days and followed by dark-adapted period of 18 h. As the severity of retinal phototoxicity in rodents depends on the circadian rhythm (Vaughan et al., 2002) the light damage experiments were always performed in the early morning according to previously described protocols (Braunger et al., 2013a; Boneva et al., 2016). Briefly, mice were isolated placed in reflective cages and exposed to diffuse cool, white fluorescent light coming from the top of the cage for the constant LD model with an intensity of 1000 lux for 24 h and for the acute LD model with 5000 lux for 0.5 h. The average light intensity was measured on the cage floor. After light exposure, mice were allowed to recover first for 6 h in dim light, and then brought back to normal cyclic light conditions until sacrifice. The effects of LD were evaluated 1 and 3 days after light exposure (Supplementary Figures S1A–C).

### Antibodies

Used antibodies are listed in Supplementary Table S1. The polyclonal anti-mouse CFP serum was generated by sequential immunizations of rats with recombinant murine CFP as described previously (Pauly et al., 2014).

### Terminal dUTP Transferase Mediated Nicked-End Labeling (TUNEL)

Apoptotic cell death was analyzed by TUNEL labeling using the Apoptosis Detection System (DeadEnd Fluorometric TUNEL, Promega). The light exposed and control retinae were investigated following manufacturers’ instructions and protocols published previously (Braunger et al., 2013b, 2015; Kugler et al., 2015). For quantitative analysis, the number of TUNEL-positive nuclei in mid-horizontal sections throughout the entire retina was counted and normalized to the area of the ONL. Indicating ONL cell density DAPI-positive cells were counted within central retina (100 μm area).

### Gene Expression Analyses

One eye from each animal was enucleated and hemisected for total RNA extraction. To this end, RPE/choroid and retina were removed from eyecups and immediately deposited in RNA stabilizer (RNAlater, Qiagen, Hamburg, Germany). RNA was isolated using *Nucleospin* RNA/Protein Kit (Macherey-Nagel, Düren, Germany) and reverse-transcribed to cDNA using *QuantiTect* Reverse Transcription Kit (Qiagen, Hamburg, Germany) according to the manufacturer’s protocol. Mouse liver cDNA was prepared equally.

PCR was performed to validate mouse complement gene-specific primer pairs (Supplementary Table S2) using the following conditions: 94°C for 30 s, 60°C for 30 s, 72°C for 30 s, 33 cycles. Amplification products were analyzed using 2% agarose gels (Supplementary Figure S2), fluorescent bands were excised and amplified cDNA was purified after manufacturer’s instructions (*Nucleospin* PCR and Gel Clean-up Kit, Macherey-Nagel, Düren, Germany). GeneArt (Thermo Fisher Scientific, Braunschweig, Germany) performed sequencing of amplicons (Supplementary Table S2).

Quantitative real-time PCR (qRT-PCR) was performed on a Rotor-Gene Q PCR cycler (Qiagen, Hamburg, Germany) using mouse gene-specific primer pairs (Supplementary Table S2) and Rotor Gene Sybr green PCR Kit (95°C for 5 min, 40 cycle with 95°C for 5 s and 60°C for 10 s; Qiagen, Hamburg, Germany). A relative gene expression was calculated using mRNA levels of *actin* as a housekeeper with the ΔCT method (ΔCT = C_T_ *gene of interest* - C_T_ *actin*) (**Table 1**) for normalization as well as with the 2^-ΔΔCT^ method [fold change = 2^-(ΔCT treated-ΔCT untreated)^] for comparison of treated and untreated mice (**Figures 2**, **3** and Supplementary Figures S1, S3; Schmittgen and Livak, 2008).

**Table 1:** Local expression of complement factors in the healthy albino retina and RPE/choroid.

Factor	Main pathway	Main function	mRNA [Δ CT actin]^a^				Protein [% GAPDH signal]^b^			
				
			Retinal	RPE/choroid	*P*-value	Fold diff.	kDa	Retina	RPE/choroid	Fold diff.
C1s	Classical	Activating protease binding to C1q	8.5±0.6	**6.2±0.4**	**<0.0001**	**1.4**	140	–	**17±8**	
							52	**31±15**	6±1	5
CFB	Alternative	Component of alternative C3-/C5-convertase	13.9±1.3	12.8±0.9	0.13	1	105	–	60±22	
MBL-A	Lectin	Activating sugar binding molecule	18.9^c^	20±0.5^c^	n.d.	n.d.	74	50±10	–	–
							37	64±13	74±47	1.1
MASP-1	Lectin	Activating protease binding to MBL and ficolin	17.6±0.2^c^	15.8±2^c^	0.17	1.1	n.d.	n.d.	n.d.	n.d.
C3	Central	Component of alternative C3- and all C5-convertases	6.3±0.5	**3.7±1.6**	**0.003**	**1.7**	∼120	1±1	**34±18^d^**	34
							∼65	0.3±0.5	**50±22^d^**	50
							∼45	3±3	**10±4^d^**	3
C4	Classical/lectin	Component of classical/lectin C3-convertase	7±0.5	6.7±1	0.62	1	160	–	33±27	
C9	Terminal	Component of membrane attack complex	17.8±0.9	18.6±3.5^c^	0.61	1	<170	36±9	**151±97**	4
CFP	Alternative	Stabilizer of alternative C3-convertase	**7.3±0.4**	9.9±0.7	**<0.0001**	**1.4**	55	108±46	142±111	1.3


*^a^Determined by quantitative real-time PCR, ^b^detected in Western blot, ^c^high CT-values: not all tested tissues were positive for gene expression (see **Figures 2**, **3** for details), ^d^C3d immunolabeling confirmed increased C3 protein content in the RPE/choroid (**Figure 5**); means ± standard deviations of six mice; n.d. not determined; unpaired *t*-test.*

### Western Blot

Protein isolation of RPE/choroid and retina (one eye from each animal) was performed with *Nucleospin* RNA/Protein Kit (Macherey-Nagel, Düren, Germany). Samples were separated on a reducing 10% SDS-PAGE and transferred onto polyvinylidene difluoride membranes. Membranes were soaked in blocking solution [5% BSA/PBS-T (PBS, 0.1% Tween 20), 1 h] and subsequently incubated with primary antibodies diluted in blocking solution (Supplementary Table S1, overnight, 4°C). After washing steps, membranes were treated with detection antibodies diluted in blocking solution (Supplementary Table S1, 1 h) and developed with Lumi-Light blotting substrate (Roche Diagnostics GmbH, Mannheim, Germany) or WesternSure PREMIUM Chemiluminescent Substrate (LI-COR, Bad Homburg, Germany).

### Immunostainings

Retinae were immersion-fixed (4% paraformaldehyde, 4 h) and embedded in paraffin. Six micrometer thick sections were used. For epitope retrieval paraffin sections were deparaffinized and heated in citrate-buffer pH 6 (1 h in a steamer, #ZUC028-500, Zytomed, Berlin, Germany). For C3d and glial fibrillary acidic protein (GFAP) analysis unspecific bindings were blocked (30 min, #ZUC007-100, Zytomed, Berlin, Germany) and primary antibodies were incubated overnight at 4°C in antibody diluent (#ZUC025-500, Zytomed, Berlin, Germany). Sections were washed (0.1% Tween 20/PBS) and incubated with secondary antibodies in antibody diluent (50 min). Cell nuclei were labeled with DAPI/Hoechst 33342 (1:1000; #H1399, Thermo Fisher Scientific, Braunschweig, Germany). After several washes (0.1% Tween 20/PBS, PBS) the slices were embedded in fluorescent mounting medium (#S302380-2, Dako, Glostrup, Denmark). For the staining of the ionized calcium binding adaptor molecule 1 (Iba-1) we used the Zytochem Plus AP Polymer Kit (#POLAP-006, Zytomed, Berlin, Germany) according to the manufacturer’s protocol. Brief, sections were blocked with 3% H_2_O_2_ (10 min) and blocking solution (10 min). Primary antibody and DAPI/Hoechst 33342 were incubated in antibody solution (4 h). Detection was performed using AP-polymer-rabbit (1 h) and permanent AP red (#ZUC001-125, Zytomed, Berlin, Germany). Every incubation step was followed by washing (0.1% Tween20/PBS). For C5b-C9 staining, the sections were blocked with 5% bovine serum albumin (BSA, Roth Karlsruhe, Germany) in 0.1 M phosphate buffer (1 h) and incubated with anti-C5b-C9 in blocking solution (4°C overnight). Detection was performed using a biotinylated anti-rabbit IgG, Alexa 488 conjugated streptavidin and DAPI counterstaining in fluorescent mounting medium. Images were acquired using confocal microscopy (VisiScope, Visitron System, Puchheim, Germany).

### C3a/C5a ELISA

Systemic complement activation was analyzed in serum. Maxisorp microtiter plates (Nalgene Nunc, Penfield, NY, United States) were coated with anti-mouse C3a and anti-mouse C5a (phosphate buffer pH 6.5 (C3a) or carbonate buffer pH 9.5 (C5a), 4°C, overnight) (Supplementary Table S1), respectively. After blocking [2% skim milk in PBS-T (C3a) or 10% FCS in PBS (C5a), 1 h], mouse sera (1:5–1:50), native mouse C3a and recombinant mouse C5a (for the standard curve 1–100 ng/mL, BD Bioscience) in sample buffer [0.1 mg/mL Nafamostat Mesylate (Futhan, TCI, Eschborn, Germany), 10 mM EDTA in PBS] were incubated (2 h). Each incubation step was followed by three consecutive washing steps (PBS-T). Detection was performed with anti-mouse C3a-biotin- and anti-mouse C5a-biotin-antibody (PBS, 1 h) (Supplementary Table S1) and a final incubation with streptavidin-HRP (1:5000 in PBS). Signal was developed using 3,3′,5,5′-tetramethylbenzidine (TMB, Seramun Diagnostica GmbH, Heidesee/Wolzig, Germany). Optical density (absorption) was measured at 450 nm.

### Software and Statistical Analysis

Data were statistically analyzed using GraphPad Prism 5 (GraphPad Software, San Diego, CA, United States). Western blot and microscopy pictures were evaluated using ImageJ (Schneider et al., 2012).

## Results

### Light-Treatment Caused Damage in the Retina

**FIGURE 1:**
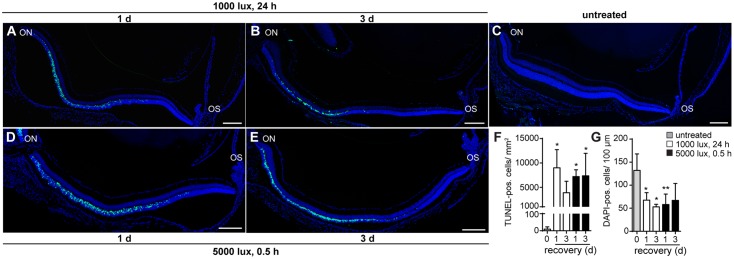
Cell death occurs in the ONL 1 day post-light treatment. Detection of cell death in retinae of mice either treated with **(A,B)** 1000 lux (24 h) or **(D,E)** 5000 lux (0.5 h) was performed by TUNEL- (green) and DAPI-staining (dark blue) of cell nuclei. **(C)** Untreated, Balb/c mice did not show TUNEL-positive cells in the retina. Albino mice treated with light showed apoptotic TUNEL positive cells (green) in the ONL **(A,D)** 1 day and **(B,E)** 3 days after light exposure. There was a significant increase in numbers of **(F)** TUNEL positive cells and a significant decrease in numbers of **(G)** DAPI positive cells following light exposure, however, no significant differences in the numbers of **(F)** TUNEL- and **(G)** DAPI-positive cells regarding the light treatment regimen was observed. Scale bars, 200 μm. ^∗^0.01 < *P* < 0.05, ^∗∗^0.001 < *P* < 0.01 (ordinary one-way ANOVA, Dunnett’s multiple comparisons test, with a single pooled variance) ON, optic nerve; OS, ora serrata.

Photoreceptor cell death is a hallmark in LD (Grimm and Remé, 2013). In order to determine whether constant (1000 lux, 24 h) or acute (5000 lux, 0.5 h) white light treatment differ in the progression of LD, we counted TUNEL-positive cells indicating apoptosis (**Figures 1A–F**), and DAPI-positive cell nuclei (**Figures 1A–E,G**), displaying photoreceptor density in the ONL. Cell apoptosis occurred in the ONL of mice treated with white light (**Figures 1A–F**) irrespective of the light intensities. TUNEL-positive cells were observed in the central region of mouse retinae 1 and 3 days post-light treatment (**Figures 1A,B,D,E,F**). Overall cell loss was indicated by a reduced number of DAPI-positive cells in the ONL after both light treatments (**Figures 1A,B,D,E,G**). Reactivity of retinal cells to light stimuli was proven by increased expression levels of LD marker *lif1* and *gfap* in the retina 1 and 3 days post-light-treatment (Supplementary Figures S1D–F).

### Complement Factors Were Locally Expressed in the Untreated Albino Mouse Eye

**FIGURE 2:**
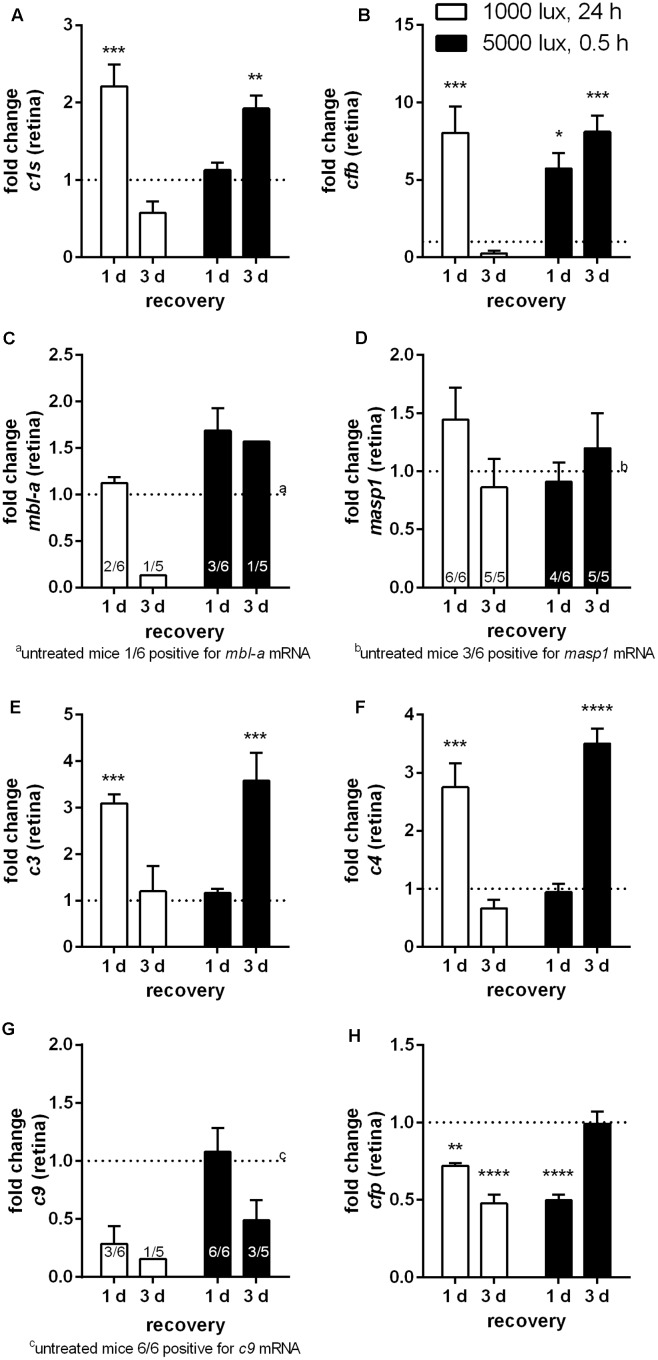
Retinal transcription of complement factors changed dependent on the recovery time after different light treatment regimens. mRNA expression was analyzed in the retinae of mice treated either with constant (1000 lux, 24 h; white bars) or acute white light (5000 lux, 0.5 h; black bars) and compared to untreated controls. **(A)** *c1s*, **(B)** *cfb*, **(C)** *mbl-a*, **(D)** *masp-1*, **(E)** *c3*, **(F)** *c4*, **(G)** *c9*, and **(H)** *cfp* mRNA was determined. **(A)** *C1s*, **(B)** *cfb*, **(E)** *c3*, and **(F)** *c4* mRNA was significantly elevated either at 1 day following constant (white column) or at 3 days following acute (black column) light treatment. **(G,H)** *C9* and *cfp* mRNA expression was decreased following LD. Data represent mean values + standard error of the mean. **(C)** *mbl-a*, **(D)** *masp-1* and **(G)** *c9* mRNA was partly detected. Positive animals were indicated as small numbers in the bars. Mean of untreated control shown as dotted line. ^∗^0.01 < *P* < 0.05, ^∗∗^0.001 < *P* < 0.01, ^∗∗∗^0.0001 < *P* < 0.001, and ^∗∗∗∗^*P* < 0.0001 (ordinary one-way ANOVA, Dunnett’s multiple comparisons test, with a single pooled variance).

**FIGURE 3:**
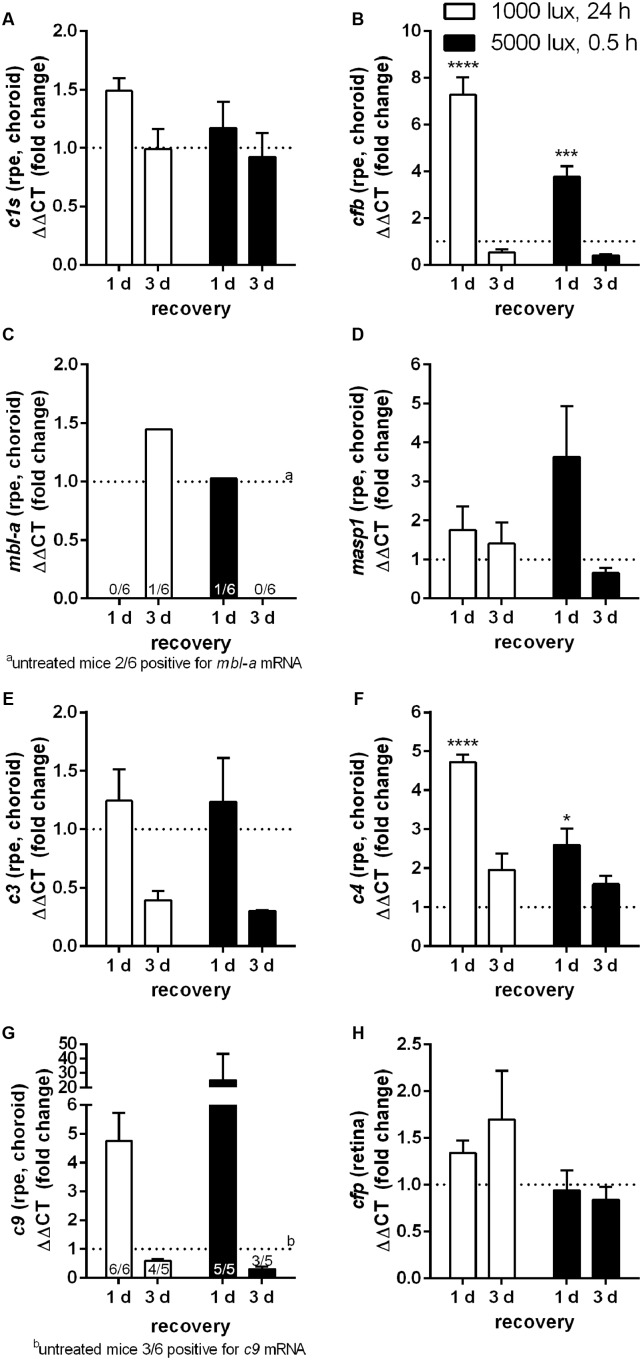
mRNA expression of complement factors in the RPE/choroid-complex is increased at 1 day following constant and acute LD. mRNA expression was analyzed in the RPE/choroid of mice treated either with constant (1000 lux, 24 h; white bars) or acute white light (5000 lux, 0.5 h; black bars) and compared to untreated controls. **(A)** *c1s*, **(B)** *cfb*, **(C)** *mbl-a*, **(D)** *masp-1*, **(E)** *c3*, **(F)** *c4*, **(G)** *c9*, and **(H)** *cfp* mRNA was determined. **(A)** *C1s*, **(B)** *cfb*, **(E)** *c3*, **(F)** *c4*, and **(G)** *c9* mRNA was elevated at 1 day post-light treatment. mRNA expression decreased 3 days following LD. **(C)** *mbl-a* and **(G)** *c9* mRNA was partly detected. Positive animals were indicated as small numbers in the bars. Data represent mean values + standard error of the mean. Mean of untreated control shown as dotted line. ^∗^0.01 < *P* < 0.05, ^∗∗∗^0.0001 < *P* < 0.001, ^∗∗∗∗^*P* < 0.0001 (ordinary one-way ANOVA, Dunnett’s multiple comparisons test, with a single pooled variance).

The aim of the study was to evaluate the role of the local complement system in LD. First, we compared complement factor expression on mRNA and protein level in the retina and RPE/choroid of untreated control albino mice (**Table 1**). Complement factors *c1s*, *cfb*, *c3*, *c4* and *cfp* mRNA were constantly expressed in the retina and RPE/choroid of untreated Balb/c mice (**Table 1**). We determined a significantly higher expression level for *c1s* and *c3* mRNA in the RPE/choroid compared to the retina (**Table 1**) and vice versa a higher expression of *cfp* mRNA in the retina than in the RPE/choroid (**Table 1**). There was no significant difference in the tissue-specific expression of *cfb* and *c4* in the eye (**Table 1**). *C9* mRNA and complement factors of the lectin pathway, *mbl-a* and *masp-1*, were detected only in a few animals of the control group (**Table 1** and **Figures 2**, **3**).

**FIGURE 4:**
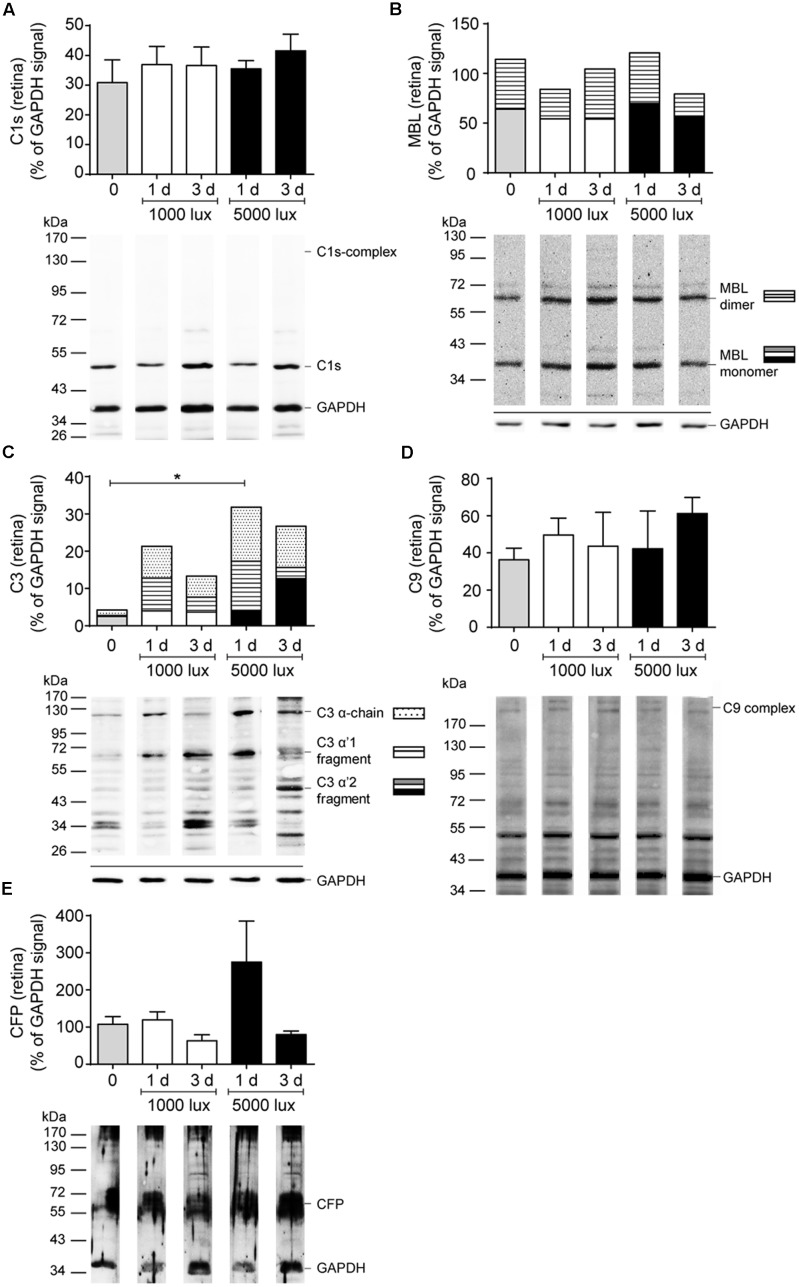
Complement protein expression in the retina showed increased C3 deposition after LD. Complement protein expression of **(A)** C1s (see also Supplementary Figure S6A), **(B)** MBL (see also Supplementary Figure S6C), **(C)** C3 (see also Supplementary Figure S6D), **(D)** C9 (see also Supplementary Figure S6B) and **(E)** CFP (see also Supplementary Figure S6E) in the retinae of mice either treated with constant (1000 lux, 24 h; white bars) or acute white light (5000 lux, 0.5 h; black bars) was compared to untreated controls (gray bars) in Western blot. **(B,C)** Stacked bars correspond to different molecular weights of protein fragments. Data represent mean percentages of the GAPDH control signal of 4–6 mice of each treatment group. Exemplarily Western blots are shown for complement protein detection in one mouse tissue, respectively (for entire blots see Supplementary Figure S6). Blots were sequentially developed (Supplementary Figure S4). CFB and C4 were not detected in retinae. ^∗^0.01 < *P* < 0.05 (ordinary one-way ANOVA, Dunnett’s multiple comparisons test, with a single pooled variance).

**FIGURE 5:**
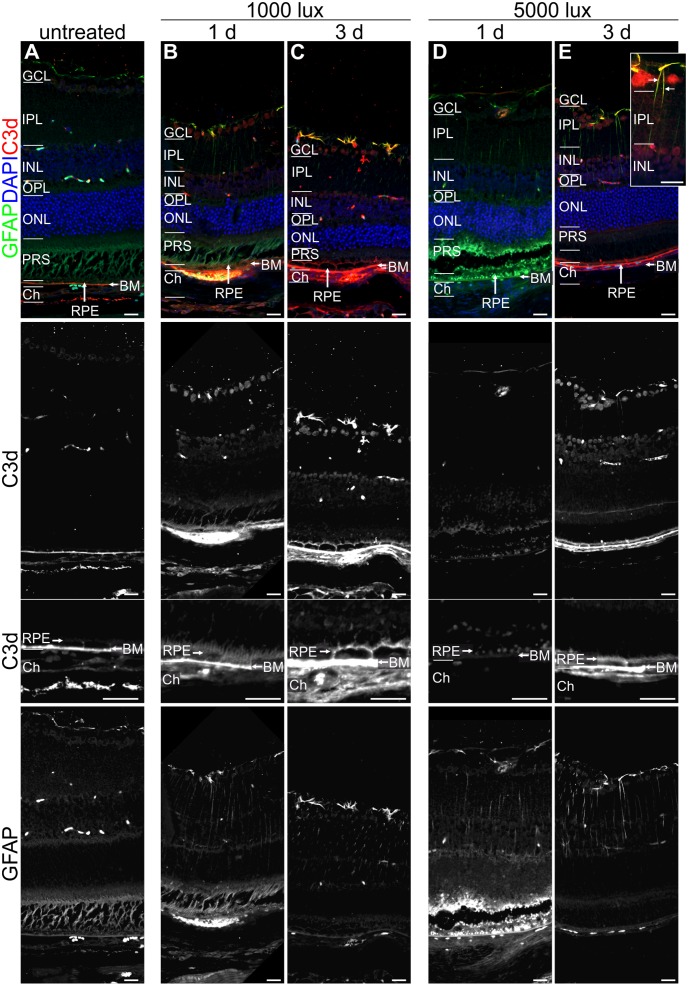
Reactive Müller cells, astrocytes and RPE are co-localized with C3d in LD retinae. Immunolabelled mouse eye sections for C3d (red) and GFAP (green) showed a LD-dependent Müller cell and astrocyte activation (GFAP, green) as well as complement activation (C3d, red), irrespective of light intensity and recovery time. **(A)** Untreated mouse retinae were negative for gliosis and showed a distinct C3d-dependent staining of Bruch’s membrane. **(B–E)** Retinae treated with bright light depicted an increased GFAP-staining of Müller cells and astrocytes which was partly co-localized with C3d staining in the retina. C3d staining at the RPE/Bruch’s membrane was increased after light treatment and additionally deposited at the surface of RPE cells. Retinal layers from the top to the bottom: GCL, ganglion cell layer; IPL, inner plexiform layer; INL, inner nuclear layer; OPL, outer plexiform layer; ONL, outer nuclear layer; PRS, photoreceptor segments; RPE, retinal pigment epithelium; BM, Bruch’s membrane; Ch, choroid. Scale bars, 20 μm.

**FIGURE 6:**
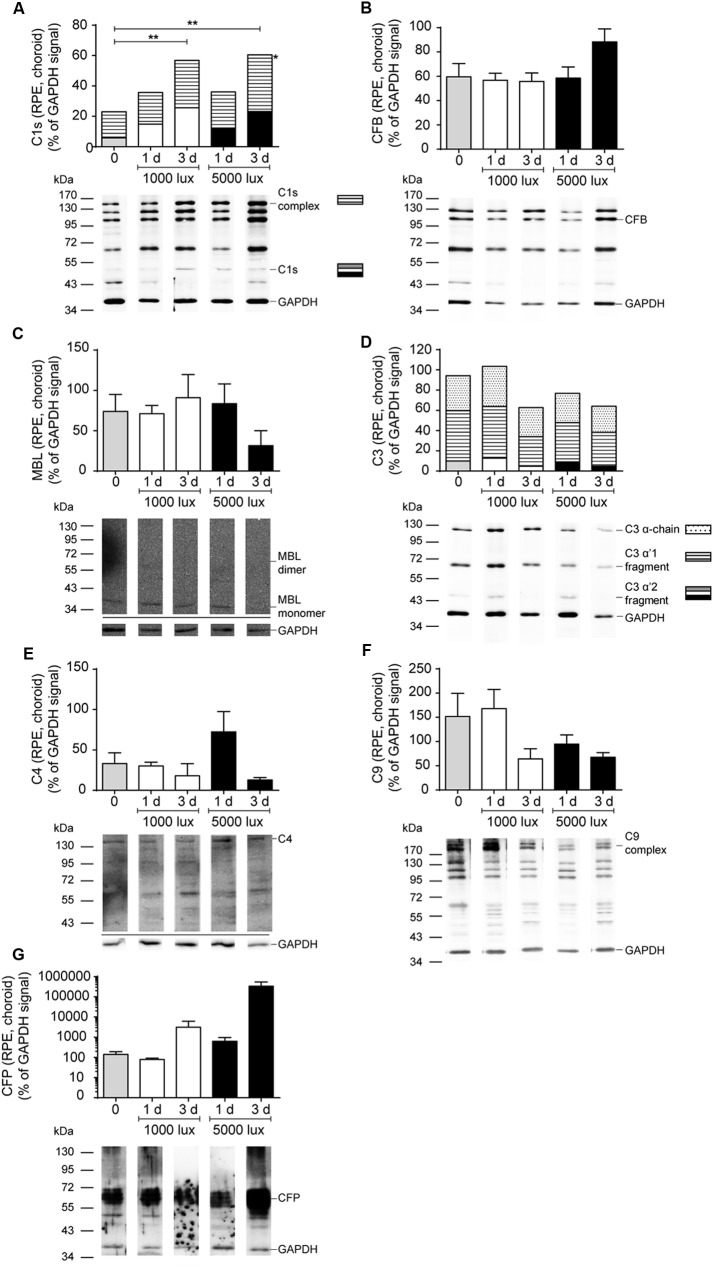
Increased C1s-complex and decreased C9-complex deposition in the RPE/choroid after LD. Complement protein expression of **(A)** C1s (see also Supplementary Figure S7D), **(B)** CFB (see also Supplementary Figure S7C), **(C)** MBL (see also Supplementary Figure S7F), **(D)** C3 (see also Supplementary Figure S7A), **(E)** C4 (see also Supplementary Figure S7H), **(F)** C9 (see also Supplementary Figure S7E), and **(G)** CFP (see also Supplementary Figure S7G) in the RPE/choroid of mice either treated with constant (1000 lux, 24 h; white bars) or acute white light (5000 lux, 0.5 h; black bars) was compared to untreated controls (gray bars) in Western blot. **(A,D)** Stacked bars correspond to different molecular weights of protein fragments. Data represent mean percentages of the GAPDH control signal of 4–6 mice of each treatment group. Exemplarily Western blots are shown for complement protein detection in one mouse tissue, respectively (for entire blots see Supplementary Figure S7). Blots were sequentially developed (Supplementary Figure S4). ^∗∗^0.001 < *P* < 0.01 (ordinary one-way ANOVA, Dunnett’s multiple comparisons test, with a single pooled variance).

All tested complement factors were found on protein level in the RPE/choroid (**Table 1**). In general, the retina was less positive for complement deposition than the RPE/choroid as we observed no CFB or C4 protein and low C3 protein levels in the untreated retina (**Table 1** and **Figures 4**–**6**). The increased tissue-specific mRNA expression for *c3* in the RPE/choroid (**Table 1**) was confirmed by increased protein detection in Western blots (**Table 1** and **Figures 4C**, **6D**) and immunohistochemistry (**Figure 5**). Higher molecular weight complexes containing either C1s or C9 showed also a 4–5-fold enhanced protein detection signal in the RPE/choroid than in the retina (**Table 1**, **Figures 4A,D**, **6A,F**, and Supplementary Figures S6A,B, S7D,E).

### White Light Increased *c1s*, *cfb*, *c3*, and *c4* Expression in the Retina Dependent on the Recovery Time, But Decreased *cfp* and *c9* Expression

Complement activation was previously described as a cause for LD (Rohrer et al., 2007; Rutar et al., 2011). We asked the question, whether there is a difference in complement factor expression following constant (1000 lux, 24 h) or acute (5000 lux, 0.5 h) LD in the eye (**Figures 2**, **3**). We identified a significant increase of *c1s*, *cfb*, *c3* and *c4* mRNA expression in the retina following both light treatment regimens (**Figures 2A,B,E,F**). Interestingly, there was a difference in the reaction regarding the recovery time after light treatment. Mice treated with 1000 lux for 24 h (constant) showed an enhanced complement gene expression in the retina after 1 day recovery (**Figure 2**, white bars), which correlated with the time-dependent apoptosis in the ONL (**Figure 1F**). In contrast, mice treated with 5000 lux for 0.5 h (acute) showed a change in *c1s*, *cfb*, *c3* and *c4* expression in the retina mainly 3 days after light illumination (**Figure 2**, black bars).

The terminal complement factor *c9* and the alternative cascade stabilizer *cfp* were constantly expressed in the healthy retina (**Table 1**), but the expression signals were decreased in the light treated animals (**Figures 2G,H**). *Cfp* mRNA expression was promptly regulated and a significant change was detected as early as 1 day post-treatment in both models (**Figure 2H**).

### Complement Factor Expression in the RPE/Choroid Changed Independently from the Light Intensity and Increased at 1 Day But Decreased at 3 Days Post-LD

The complement factor mRNA expression in the RPE/choroid correlated in the constant light (1000 lux, 24 h) treated animals, for most of the analyzed factors (exception: *c9, cfp*), with the retinal expression pattern (white bars, **Figures 2**, **3**). We observed a significant increase of mRNA for *cfb* and *c4* (**Figures 3B,F**) as well as an elevated signal for *c1s*, *c3* and *c9* at 1 day compared to 3 days post-LD (**Figures 3A,E,G**). The mice treated with acute light (5000 lux, 0.5 h) showed a comparable expression as the constant light treated animals in the RPE/choroid (**Figure 3**). This was in contrast to the retinal expression pattern in the acute group, where we observed an increased mRNA expression only 3 days after LD but not immediately 1 day post-LD (black bars, **Figures 2**, **3**). The complement factor expression in the RPE/choroid decreased at 3 days post-LD in both models (**Figure 3**). We emphasize that the gene expression levels for *cfb* and *c3* even dropped below the normal expression levels of untreated mice (**Figures 3B,E**).

### Similar Regulation of Complement Factors at mRNA Levels in Acute and Constant LD

The described complement gene expression studies (**Figures 2**, **3**) suggested a pattern for the regulation of complement gene expression following LD. Indeed, when we plotted the gene expression profiles of all tested genes in one graph corresponding to the used light intensities and tested tissues (Supplementary Figure S3), we found two different mRNA expression profiles. On the one hand, a similar expression pattern in the RPE/choroid complex following constant (1000 lux, 24 h) and acute (5000 lux, 0.5 h) LD as well as in the retina after constant LD (Supplementary Figures S3A,C,D): Complement mRNA expression decreased from 1 day recovery to 3 days recovery. On the other hand, this progress of complement expression appeared to lag behind in the retinae of animals treated with 5000 lux (Supplementary Figure S3B): All complement factors (except *cfb*) showed no change in expression level after 1 day recovery in mice treated with 5000 lux compared to the control group. However, in contrast to the 1000 lux treated animals the mice showed an increase of complement expression in the retina at 3 days (except *c9*) (Supplementary Figure S3B).

### C3 Deposition Was Increased after LD

Further, we investigated if the mRNA expression of complement genes corresponded to protein detection in the eye (**Figures 4–6** and Supplementary Figures S4, S6, S7). The only significant changes in the retina after light treatment were observed in regard to C3-the central complement protein (**Figures 4C**, **5** and Supplementary Figure S6D). C3 protein concentrations were elevated in all light-treated mice retinae compared to the control in Western blots, irrespectively of the recovery time (**Figure 4C** and Supplementary Figure S6D). C3d deposition, which is a cleavage product of C3b, was also increased in immunostainings of tissue sections following acute and constant LD (**Figure 5**). C3d-staining in the LD-retinae co-localized with GFAP-reactive Müller cells and astrocytes (**Figure 5**). The increased C3 protein detection in the retina correlated with increased *c3* mRNA expression in the retina after LD (**Figure 2E**), except that the *c3*-transcription level in the acute model did not change after 1 day recovery (**Figure 2E**).

The trend for diminished *c3* mRNA expression in the RPE/choroid complex 3 days after constant and acute LD (**Figure 3E**) corresponded to decreased C3 protein detection in Western blots in the RPE/choroid (**Figure 6D** and Supplementary Figure S7A). Especially, the C3-alpha chain fragment (45 kDa) was missing after 3 days in the RPE/choroid-complex (**Figure 6D** and Supplementary Figure S7A). In contrast, we observed an increased C3d immunostaining in the RPE/choroid after LD (**Figures 5B–E**). C3d was deposited at the surface of the RPE-cells following both light regimens (**Figures 5B–E**). The differences in C3d-detection in Western Blots and immunostainings could be explained by the different specificities and affinities of the antibodies used here: the anti-C3 antibody in the Western Blots detected C3 irrespective of its activation pattern (**Figure 6D** and Supplementary Figure S7A) and in the immunostaining the antibody showed a selective specificity against the C3d activation product (**Figure 5**).

### Classical Complement Pathway Initiator C1s-Protein Complex Accumulated in RPE/Choroid after LD

C1s is a protease, which acts in a high molecular weight protein complex with C1r and C1q. Western blots from the RPE/choroid of LD mice showed a significant increase of the C1s-complex (140 kDa) 3 days post-light treatment (**Figure 6A** and Supplementary Figure S7D). The 2-times higher C1s-complex signal in the RPE/choroid did not completely overlap with an elevated local *c1s* mRNA expression following light-treatment but could be a result of a delayed increase of C1s protein expression compared to its transcript in the retina and RPE/choroid at 1 day after constant and acute light illumination (**Figures 2A**, **3A**). We did not observe a change in C1s protein deposition in the retina (**Figure 4A** and Supplementary Figure S6A).

### LD Resulted in Reduced Soluble C9-Complex Concentrations in the RPE/Choroid and Increased C5b-C9 Deposition in the Retina

C9 is the central protein of the terminal pathway of the complement system and forms the membrane attack complex together with C5b-C8. Soluble C9 protein detection in untreated mice was 4-fold higher in the RPE/choroid than in retina (**Table 1**, **Figures 4D**, **6F**, and Supplementary Figures S6B, S7E). LD resulted in decreased soluble C9 levels at 3 days in the RPE/choroid in both treatment regimens compared to the control (**Figure 6F** and Supplementary Figure S7E). This correlated with decreased *c9* mRNA expression in the RPE/choroid following constant and acute white light-treatment after 3 days recovery (**Figure 3G**). While the concentration of the terminal complement membrane attack complex, consisting of C5b-C9, was enhanced after light-treatment in the retinae (Supplementary Figure S5).

### Müller Cells, Astrocytes, and Microglia/Macrophages Were Activated 1 Day after Light Treatment

**FIGURE 7:**
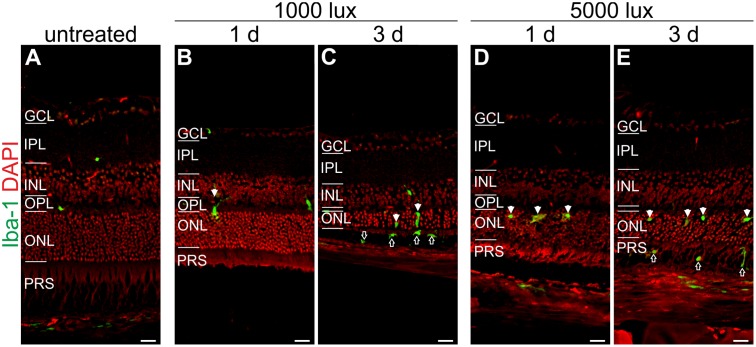
Microglia/macrophage cells migrate at 1 day after LD in the ONL and at 3 days in the PRS. Immunoreactivity for Iba-1 (green) showed microglia/macrophage distribution in **(A)** an untreated mouse retina, **(B,C)** retinae treated with 1000 lux for 24 h and **(D,E)** retinae illuminated with 5000 lux for 0.5 h. **(B,D)** Mice showed microglia/macrophage migration into the ONL at 1 day post-LD (white arrows). **(C,E)** Three days post-light treatment microglia/macrophages were located in the ONL (white arrows) and additionally in the PRS (black arrows) in both light treatment regimens. Retinal layers from the top to the bottom: GCL, ganglion cell layer; IPL, inner plexiform layer; INL, inner nuclear layer; OPL, outer plexiform layer; ONL, outer nuclear layer; PRS, photoreceptor segments; RPE, retinal pigment epithelium; Ch, choroid. Scale bars, 20 μm.

Next we asked the question whether the local, time dependent expression of complement factors after LD is associated with local cellular activity. Hence, the activity of Müller cells and astrocytes (**Figure 5**) as well as the migration of microglia/macrophages was assessed by immunostaining (**Figure 7**). GFAP-staining (a marker for gliosis) showed reactive Müller cells and astrocytes in all mice at 1 and 3 days after light treatment (**Figure 5**), but not in the untreated animals. It is of note that a co-localization of C3d and GFAP was observed in the light treated mouse retinae (**Figure 5**).

Iba-1 positive cells (a marker for microglia/macrophages) were sparsely localized in the plexiform layers and in the choroid of untreated mice (**Figure 7A**). A moderate recruitment of amoeboid microglia/macrophages into the ONL was observed after light treatment in both light regimens (white arrows, **Figures 7B–D**). An extended recovery time of 3 days after LD promoted microglia/macrophage migration into the photoreceptor layer (PRS) (black arrows, **Figures 7C,E**). We did not observe an obvious difference in the microglia/macrophage reaction pattern using constant or acute white light treatment.

### LD Resulted in Increased Systemic C3a and C5a Concentrations

**FIGURE 8:**
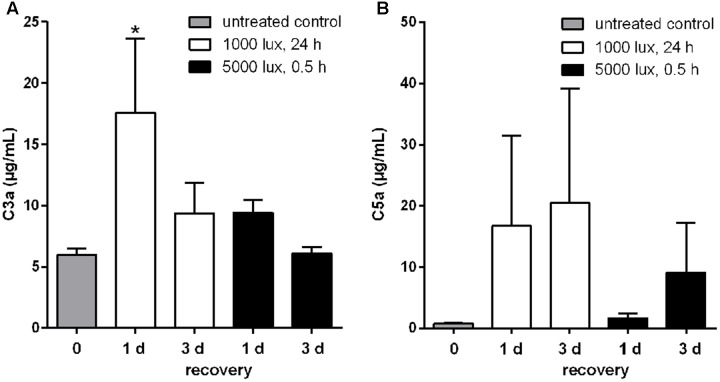
Systemic C3a and C5a concentrations were altered in LD mice. Anaphylatoxin concentrations, **(A)** C3a and **(B)** C5a, in sera of mice treated with white light were determined in ELISA. **(A)** C3a and **(B)** C5a concentrations were elevated compared to the control group, but showed a wide concentration range and standard error within the groups. **(A)** Systemic C3a concentration in mice was significantly increased 1 day post-constant light treatment (1000 lux, 24 h). Data represent mean values + standard error mean of six mice. ^∗^0.01 < *P* < 0.05 (ordinary one-way ANOVA, Dunnett’s multiple comparisons test, with a single pooled variance).

The interaction of local and systemic complement system in retinal degeneration is still under debate. We evaluated systemic complement involvement in mice after LD by determining serum concentrations of C3a and C5a, which are activation markers for all three complement pathways (**Figure 8**). The systemic reaction toward light treatment was very heterogeneous. We observed a partial increase of anaphylatoxin concentrations, but not in all animals, following LD (**Figure 8**). We identified a significant increase of C3a concentration after 1 day of constant light treatment (1000 lux, 24 h) (**Figure 8A**).

## Discussion

### Complement Factors Are Locally Expressed in the Healthy, Albino Mouse Retina

Dysregulation of the complement system in the eye is associated with retinal degeneration and damage of the blood-retinal barrier (Weber et al., 2014). However, we are still confronted with a chicken and egg problem in regard to retinal degeneration. At what time is which player activating the complement cascade in the eye? Is the systemic complement system involved, which is produced in the liver or by immune cells and then transported by the blood into the choroid? Or is the damage of the retinal blood barrier associated with locally produced complement factors by RPE cells and retinal cells? Successful treatment strategies for retinal degeneration depend on an effective on-site drug application and therefore our knowledge about regional and temporal complement involvement needs to be extended (Volz and Pauly, 2015).

In this study, we focused on eye tissue specific complement factor analysis, dissecting the retina from the RPE/choroid. In accordance with the literature we showed, that *c3* and *c1s* mRNA were mainly expressed in the RPE/choroid of healthy albino mouse eyes (Luo et al., 2011; Gemenetzi and Lotery, 2015). Conversely, *cfp* mRNA was expressed at higher amounts in the retina and the local expression levels of *cfb*, *c4* and *c9* mRNA were equivalent in both eye tissues (Luo et al., 2013; Woodell et al., 2013). In contrast, genes involved in the lectin pathway, *mbl-a* and *masp-1*, were only detected in a few control mice in retinal and RPE/choroid samples. Lou et al. described also a lack of *mbl* and low *masp* expression in the mouse eye (Luo et al., 2011).

The tested complement factors in this study were soluble, secreted proteins which were deposited and acted independently of their mRNA synthesis site. A tissue-specific correlation of mRNA detection and protein deposition revealed only for C3 and MASP-1 an overlap of similar, local mRNA and protein levels. However, we detected enhanced mRNA levels for *c1s* in the RPE/choroid but more C1s protein in the retina than in the RPE/choroid. Additionally, we determined an equivalent expression of *cfb*, *c4*, and *c9* mRNA in both tissues, but an enhanced tissue-specific protein detection either in the retina for CFB and C4 or in the RPE/choroid for C9.

In summary, we conclude that complement factors are produced by healthy eye tissues on mRNA and protein level. Therefore, local complement expression could play an important role in the modulation of the complement homeostasis in the mouse eye and should be evaluated in parallel on mRNA levels as well as on protein levels. Care has to be taken when concluding from immunolocalization of respective complement proteins to the cell types actually producing the proteins given their features as secreted proteins.

### Complement Expression Is Time-Dependently Altered in the Murine Eye as a Consequence of LD

Rohrer et al. (2007), Rutar et al. (2011), Hadziahmetovic et al. (2012), and Song et al. (2012) suggested an involvement of the complement system in the degenerative processes in LD and described an upregulation of *c3* mRNA expression 3 h after LD and with a plateau at d4–10 post-LD. We confirmed these results, describing a statistically significant difference in *c3* mRNA expression at 1 day after constant and 3 days after acute light treatment compared to untreated animals (**Figure 2E**). These previous studies did not report a decrease of *c3* mRNA expression at 3 days post-LD (Rohrer et al., 2007; Rutar et al., 2011), which was characteristic for the studied mRNA expression in the constant and acute LD models in the RPE/choroid and the constant model in the retina (**Figures 2E**, **3E** and Supplementary Figure S3). Likely reasons for the differences could be that in the study by Rohrer et al. (2007) the mice were treated with continuous white light without a low light recovery phase and in our study light treatment was used as a trigger mechanism for a maximum of 24 h. Rutar et al. (2011) used rats for LD. Perhaps LD-dependent complement expression is different in murine and rat retina. This hypothesis is supported by novel data of the same group showing, that for pigmented light treated mice *c3* mRNA expression decreases after a peak expression at d5 again in contrast to *c3* expression in rats (Rutar et al., 2011; Natoli et al., 2016)

Additionally to the central complement component C3, we report a tissue- and LD model-dependent increase of C1s mRNA and protein after LD (**Figures 2A**, **3A**, **4A**, **6A** and Supplementary Figures S6A, S7D). The upregulation of this classical pathway activation factor was also described previously for LD in rats (Rutar et al., 2011). We emphasized the function of C1s in LD by showing for the first time an elevated C1s-protein complex accumulation in the RPE/choroid complex after LD (**Figure 6A** and Supplementary Figure S7D). C1s is a protease which acts in concert with C1q and C1r and cleaves components of the classical complement pathway. This could implicate an involvement of the classical complement pathway at the retinal-blood-barrier after LD. It was already proposed that the C1 complex could be involved in retinal degeneration, since a single nucleotide polymorphism in the C1-inhibitor is associated with AMD (Ennis et al., 2008). However, a deficiency in C1q did not result in a significant difference of retinal structure following LD compared to wild-type mice (Rohrer et al., 2007).

The possible involvement of the classical (C1s) pathway in LD requires the activity of molecules downstream of the activation step to execute the full biological function of the complement system in LD. We showed in accordance with previous reports, that *c4* mRNA, a part of the classical/lectin C3/C5-convertase, is upregulated in the retina and RPE/choroid following LD (**Figures 2F**, **3F**; Rohrer et al., 2007; Rutar et al., 2011), but we could not correlate changes on protein level for C4 (**Figure 6E** and Supplementary Figure S7H), probably due to antibody detection problems. Nevertheless, this complement component should be further studied in retinal degeneration, especially in regard to the complexity of the *c4* gene locus and the association of copy number variations of the C4A gene in different disease (Grassmann et al., 2016; Sekar et al., 2016).

Interestingly, Rohrer et al. (2007) suggested an important role of the alternative complement pathway in LD as photoreceptors of complement factor D-deficient mice were significantly protected from LD. The important function of the alternative pathway in retinal degeneration was also shown in CFB-deficient mice, which were protected from retinal degeneration by laser-induced choroidal neovascularization and cigarette smoke exposure (Woodell et al., 2013; Schnabolk et al., 2015). We showed in accordance with these studies that *cfb* mRNA is strongly and promptly upregulated in the RPE/choroid and in the retina after LD, this promotes the role of CFB and the alternative pathway in LD (**Figures 2B**, **3B**), while we did not observe CFB protein deposition in the retina and no change in the RPE/choroid with the used antibodies (**Figure 6B** and Supplementary Figure S7C). CFB is cleaved into Bb and Ba during complement activation. Therefore, further studies analyzing the function of CFB in retinal damage should focus on the detection of the CFB cleavage products, Bb and Ba, in the retina to elucidate our reported discrepancy between enhanced *cfb* mRNA expression and the uncorrelated CFB full-length protein detection.

The lectin pathway is the third complement activation mechanism of the complement system and microarray studies showed a changed expression for ficolin, an initiating molecule of the lectin pathway, in rats after LD (Rutar et al., 2011). Our studies revealed a low expression of *mbl-a* and *masp-1* after LD (**Table 1**, Supplementary Figure S2, and **Figures 2C,D**, **3C,D**) and no significant change either on protein or on mRNA levels (**Figures 4B**, **6C** and Supplementary Figures S6C, S7F). However, a participation of the lectin pathway in retinal degeneration was proposed as it was shown that MBL and MASP-1 were systemically upregulated in late AMD-patients (Osthoff et al., 2015; Kim et al., 2016). Taking our results into account, it seems that previously reported effects of the lectin pathway on RPE cells and in a laser-induced neovascularization mouse model are either supported by systemic MBL and MASP or other lectin activating molecules like ficolin, than by locally expressed *mbl-a* and *masp-1* (Rohrer et al., 2011; Joseph et al., 2013).

In context of complement profiling in LD, we also aimed to analyze the role of C9, the main component of the terminal complement complex. C9 had been proposed to be relevant in laser-induced choroidal neovascularization in mice (Rohrer et al., 2011; Birke et al., 2014) and had been associated with the development of AMD (Wang et al., 2010; Seddon et al., 2013). Surprisingly, the transcription of the *c9* gene was, besides *cfp*, the only complement gene which was downregulated after LD in the retina, but immediately upregulated in the RPE/choroid (**Figures 2G**, **3G**). This is in accordance with previously shown increased deposition of terminal complement complexes at the RPE/choroid interface in mice and human age-related retinal modifications (Tomita et al., 2005; Izawa et al., 2016; Niwa et al., 2016), but it is contrary to the decreasing protein concentration of C9 in the RPE/choroid determined by Western blots in this study (**Figure 6F** and Supplementary Figure S7E). We suggest that the enhanced *c9* mRNA synthesis in the RPE/choroid following LD resulted in higher concentrations of C9 protein, which was immediately integrated into membrane pores (Supplementary Figures S5C–E) and could not be purified and detected in the used Western blot protocol (**Figures 4D**, **6F** and Supplementary Figures S6B, S7E). Therefore, the soluble C9 protein concentration was decreased following LD, but the membrane associated C9 concentration was increased after retinal damage (Tomita et al., 2005; Izawa et al., 2016; Niwa et al., 2016).

Complement activity is tightly regulated by membrane-bound and soluble complement factors. We assumed that the expression of CFP, the only known stabilizing regulator of the alternative complement pathway, would also be altered in LD. Surprisingly, *cfp* mRNA expression was not upregulated after LD like other tested complement factors (**Figures 2H**, **3H**). *Cfp* expression has not been evaluated in previous LD studies. Indeed, *cfp* expression has been shown to be quite stable *in vivo* and *in vitro* in response to several stimuli such as preeclampsia or renal tubular damage (Reis et al., 2006; Buurma et al., 2012; Nagamachi et al., 2014), while it has been described to be upregulated following shear stress in endothelium (Bongrazio et al., 2003). However, our results do not automatically imply, that CFP is not involved in degeneration, as we know from other studies that CFP deposition was positive in patient eyes with AMD (Wolf-Schnurrbusch et al., 2009) and was associated with drusen-like deposition in Alzheimer’s disease (Fonseca et al., 2011). Further studies have to be performed to decipher the expression pattern of CFP in more detail.

Interestingly, the activation pattern of the complement system (at protein level) mirrors the glial activation pattern. Similar to increasing C3 and C1s protein levels from 1 to 3 days post-treatment, the microglia/macrophages migrated into the subretinal space and got into close contact with photoreceptor segments 3 days after LD. Moreover C3d deposition on GFAP-positive Müller glia cells was only observed in retinae 3 days after LD. Specifically microglia/macrophages are known to express receptors for complement factors such as C3a (Rutar et al., 2011). In sum, this could imply that the complement activity by binding on Müller cells (e.g., C3b or C3d levels) triggers or modulates the microglial (e.g., C3a) response pattern upon tissue damage. This finding should be followed up in future studies.

## Conclusion

Our results indicate a time-dependent and tissue-specific complement modulation in close association with LD in albino mice eyes which was mostly independent of the used light intensities. In the end, the complement proteins C3, C1s and C9 were identified as putative marker proteins in the LD model to monitor disease progression, as they showed valid concentration changes in a time-dependent manner compared to control mice (**Figures 4C**, **6A,F** and Supplementary Figures S6D, S7D,E). The most significant alterations in complement protein concentration in the retina (C3) and RPE/choroid-complex (C9, C1s) after LD were observed 3 days post-treatment. The proteins were regulated in different ways: C3 and C1s were increased, but C9 was downregulated. This may indicate different functions of the proteins during retinal degeneration that need to be investigated in future studies.

Complement expression is time-dependently regulated after the initiation of cell stress in the retina and RPE/choroid. This also implies that one should monitor the eye-specific complement expression pattern after damage, when complement-manipulating reagents are tested for therapeutic reasons in the mouse eye.

## Availability of Data and Material

The datasets supporting the conclusions of this article are available at the University of Regensburg Publication Server, http://doi.org/10.5283/epub.35102

## Author Contributions

DP developed concept and designed the study. AG, BB, and DP designed experiments. NS, SS, AG, BB, and DP performed experiments. NS, BB, AG, and DP analyzed and discussed data. NS, BB, AG, and DP wrote the manuscript. All authors read and approved the final manuscript.

## Conflict of Interest Statement

The authors declare that the research was conducted in the absence of any commercial or financial relationships that could be construed as a potential conflict of interest.

## Abbreviations

CFB: complement factor B
CFP: complement factor P
ELISA: enzyme linked immunosorbent assay
LD: light-induced photoreceptor damage
MASP-1: mannose-binding protein-associated serine protease 1
MBL-A: mannose binding lectin A
ONL: outer nuclear layer
RPE: retinal pigmented epithelium
TUNEL: terminal deoxynucleotidyl transferase dUTP nick end labeling
