# Supplementary material for: Complement Components Showed a Time-Dependent Local Expression Pattern in Constant and Acute White Light-Induced Photoreceptor Damage
Source: Front Mol Neurosci. 2017 Jun 20;10:197. doi: 10.3389/fnmol.2017.00197 (PMC5476694; doi:10.3389/fnmol.2017.00197)
Supplement: Supplementary file 1 [file Data_Sheet_1.PDF]

SUPPLEMENT

Complement components showed a time-dependent local expression pattern in constant and acute white light induced-photoreceptor damage

Nicole Schäfer[1], Antje Grosche[2], Sabrina Schmitt[3], Barbara M. Braunger[3], Diana Pauly[1*]

nicole.schaefer@ukr.de, antje.grosche@ukr.de, sabrina.schmitt@ur.de, barbara.braunger@ur.de, diana.pauly@ukr.de

[1] Department of Ophthalmology, University Hospital Regensburg, Regensburg, Germany;

[2] Institute of Human Genetics, University Regensburg, Regensburg, Germany

[3] Institute of Human Anatomy and Embryology, University Regensburg, Regensburg, Germany

* Corresponding author

Supplementary: 2 Tables, 7 Figures

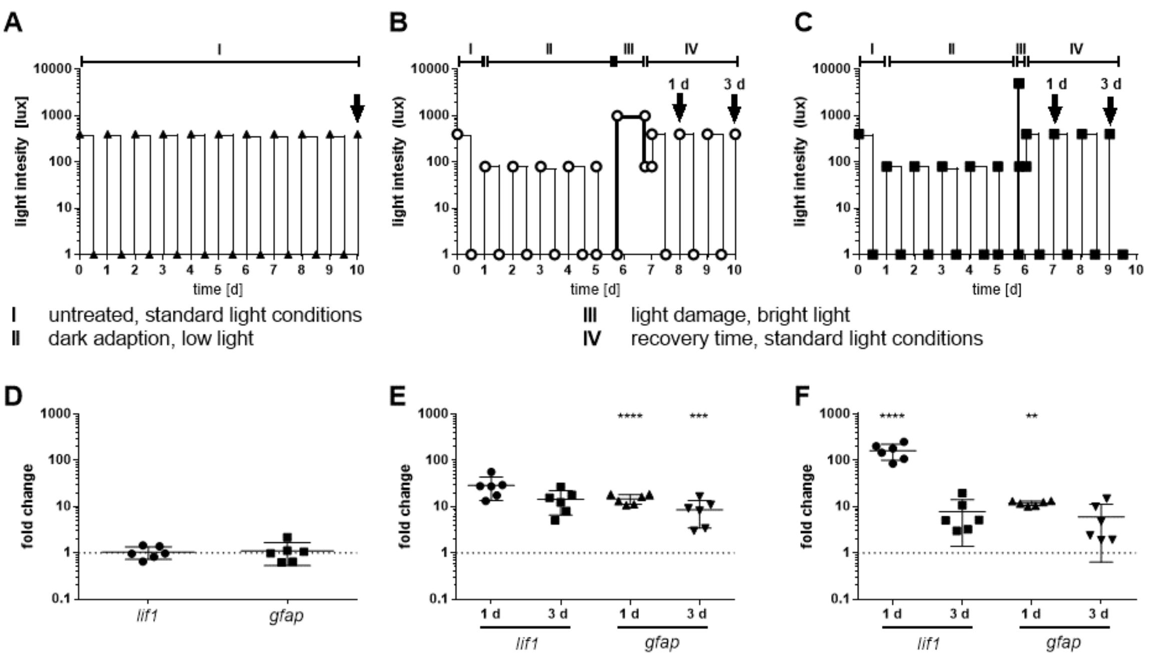

Supp. Figure 1 Light-induced photoreceptor damage (LD) after different light treatment protocols.

LD was compared in **(A)** untreated Balb/c mice, **(B)** mice treated 24 h with 1000 lux (constant model) and **(C)** mice treated 0.5 h with 5000 lux (acute model). **(A, B, C)** show times and light intensities for standard light conditions (I), dimmer regimen (II), light treatment (III) and recovery time (IV) for the different models. **(D, E, F)** Expression levels for LD marker *lif1* and *gfap* were quantified by RT-qPCR in **(D)** control mice (dotted line), **(E)** constant and **(F)** acute light treated mice. Data represent mean values ± standard error mean. **0.001<P<0.01, ***0.0001<P<0.001, ****P<0.0001 (ordinary one-way ANOVA, Dunnett's multiple comparisons test, with a single pooled variance)

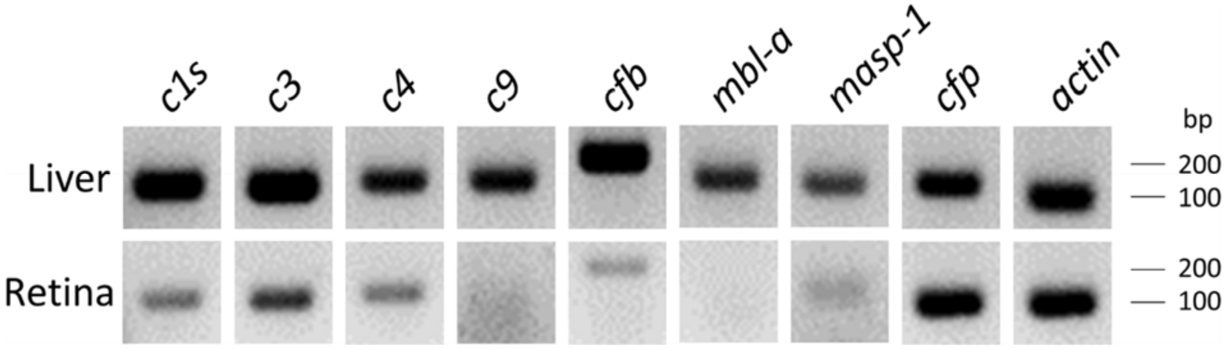

Supp. Figure 2 Amplification of complement factor fragments from liver and retina cDNA showed expected product sizes using qRT-PCR oligonucleotide primer pairs

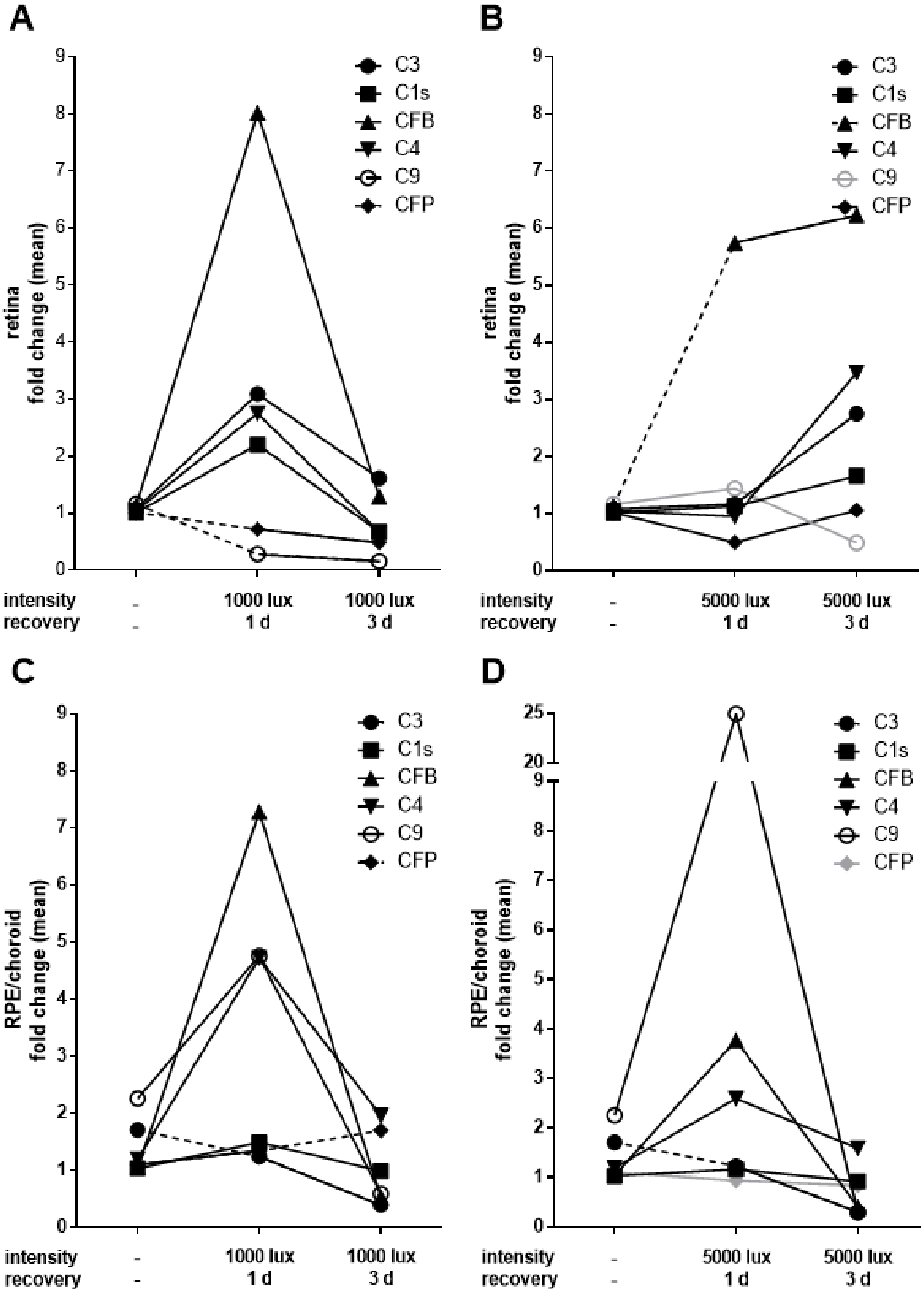
A
B
C
D
retina fold change (mean)
RPE/choroid fold change (mean)
C3
C1s
CFB
C4
C9
CFP
intensity
recovery
1000 lux
1 d
3 d
5000 lux

Supp. Figure 3 Mouse eyes revealed a time-dependent expression pattern for complement mRNA following light treatment.

Combined plotting of the respective fold change means of complement factor mRNA expression in **(A, B)** the retina and **(C, D)** the RPE/choroid of mice treated with **(A, C)** 1000 lux or **(B, D)** 5000 lux revealed similar expression patterns for complement genes after LD. Dotted lines show aberrant gene expression compared to the majority of the other genes. Grey lines in **(B)** for *c9* and **(D)** for *cfp* expression show a contrary expression pattern.

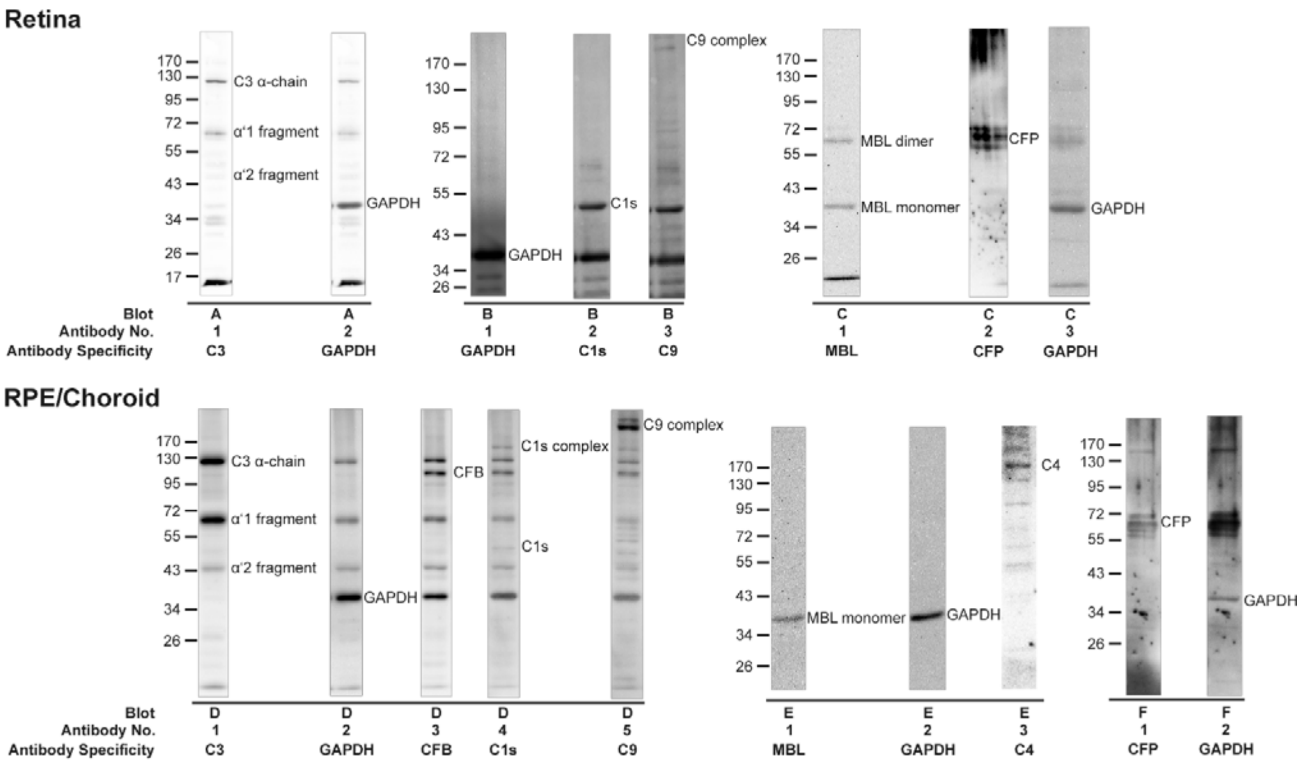

Supp. Figure 4 Western blot detection pattern for successive analyses of complement proteins in retinal and choroidal/RPE tissue.

Exemplarily shown are the entire Western blot detection pattern of 12 complement proteins from retinal and choroidal/RPE tissue of one mouse on five different membranes (blots A-F). Proteins blotted on membranes were successively incubated with 2-5 antibodies with different specificities. The anti-complement antibodies detected specifically their target proteins at appropriate protein sizes. Different C3- (blot A1) and C4-fragments (blot E3) resulted in multiple Western blot signals. C9 forms homo- and heteromers and was therefore detected at different sizes (blot D5). The used experimental conditions caused additive protein detections (1-5) on identical Western blots (A, B, C, D, E or F). Specific signals for each antibody were emphasized in Fig. 4 and 6. All retinal blots are shown in Supp. Figure 6 and all blots for the RPE/choroid are shown in Supp. Figure 7.

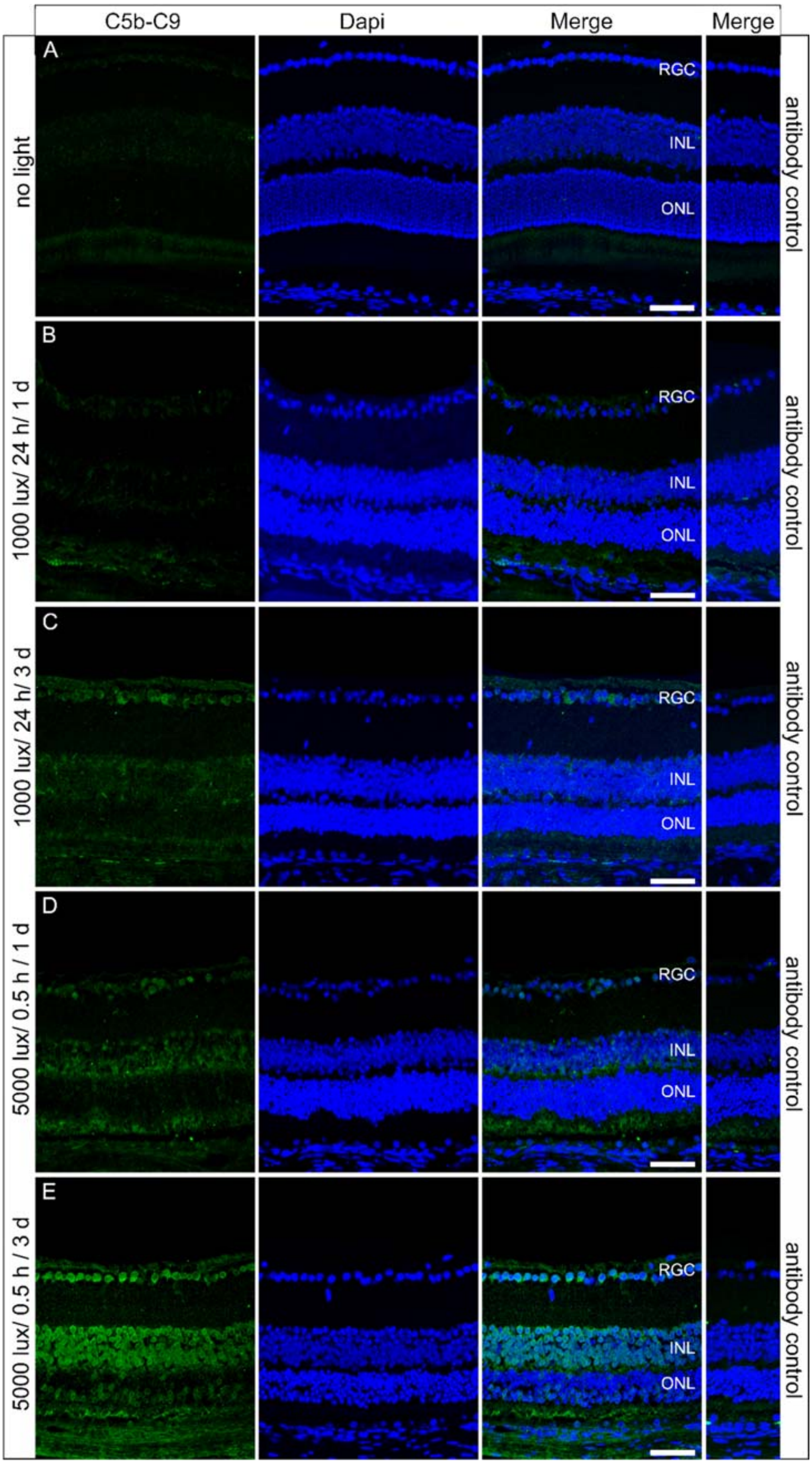
C5b-C9
Dapi
Merge
Merge
A
no light
RGC
INL
ONL
antibody control
B
1000 lux/ 24 h/ 1 d
RGC
INL
ONL
antibody control
C
1000 lux/ 24 h/ 3 d
RGC
INL
ONL
antibody control
D
5000 lux/ 0.5 h / 1 d
RGC
INL
ONL
antibody control
E
5000 lux/ 0.5 h / 3 d
RGC
INL
ONL
antibody control

50

Supp. Figure 5 C5b-C9 staining increased in the retina after light treatment.

C5b-C9 (green) was stained in **(A)** untreated and **(B-D)** white light treated murine retinae (different treatment protocols). The membrane attack complex preferentially co-localised with retinal ganglion cell (RGC, **B-E**), the inner nuclear layer (INL, **B-E**) and the photoreceptor inner segments (**D, E**) of light treated mice. Cell nuclei were stained with DAPI (blue). Retinal layers from the top to the bottom: RGC, retinal ganglion cell layer; INL, inner nuclear layer; ONL, outer nuclear layer. Scale bar 50 µm.

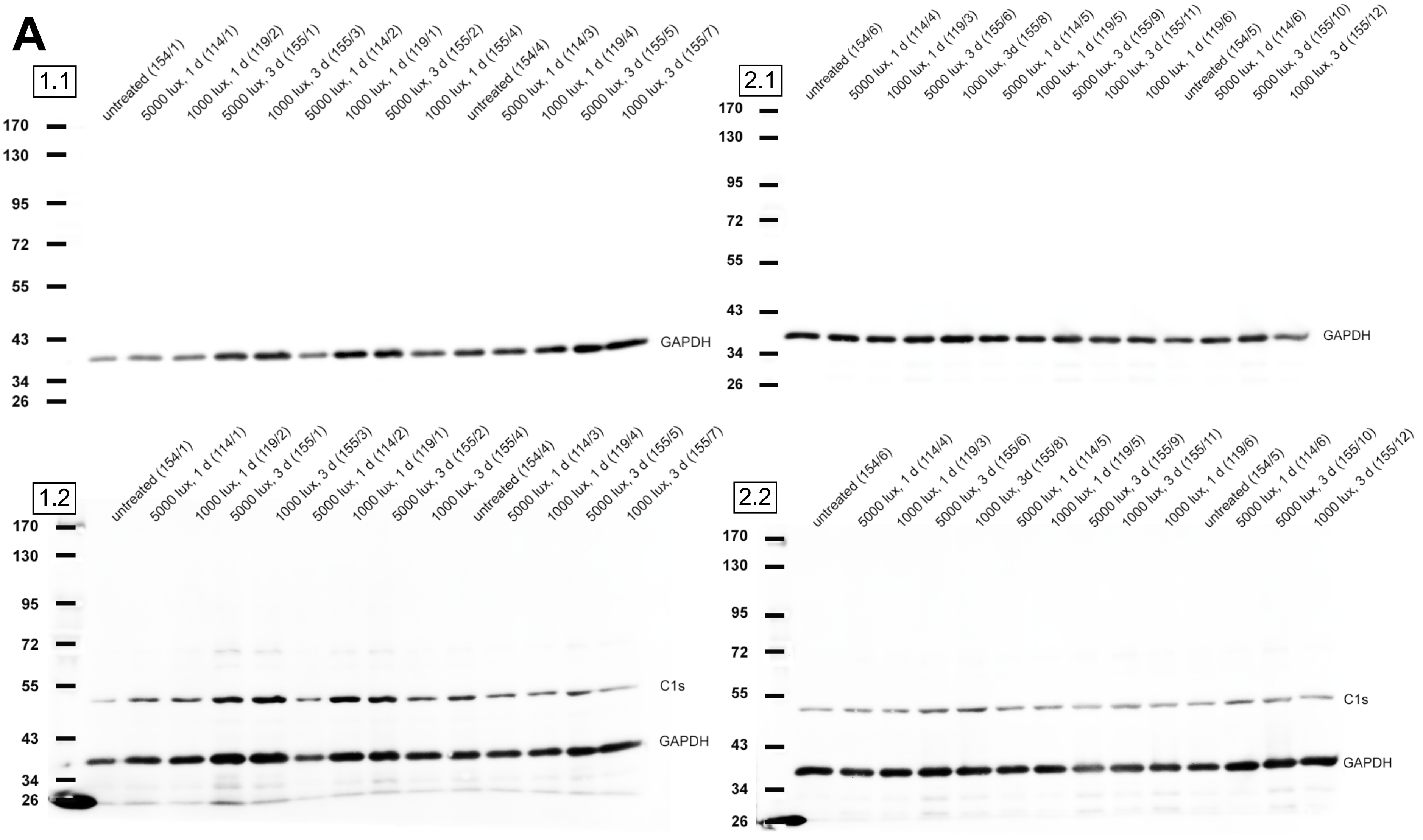
A
1.1
untreated (154/1)
5000 lux, 1 d (114/1)
1000 lux, 1 d (119/2)
5000 lux, 3 d (155/1)
1000 lux, 3 d (155/3)
5000 lux, 1 d (114/2)
1000 lux, 1 d (119/1)
5000 lux, 3 d (155/2)
1000 lux, 1 d (155/4)
untreated (154/4)
5000 lux, 1 d (114/3)
1000 lux, 1 d (119/4)
5000 lux, 3 d (155/5)
1000 lux, 3 d (155/7)
170
130
95
72
55
43
34
26
GAPDH
2.1
untreated (154/6)
5000 lux, 1 d (114/4)
1000 lux, 1 d (119/3)
5000 lux, 3 d (155/6)
1000 lux, 3d (155/8)
5000 lux, 1 d (114/5)
1000 lux, 1 d (119/5)
5000 lux, 3 d (155/9)
1000 lux, 3 d (155/11)
1000 lux, 1 d (119/6)
untreated (154/5)
5000 lux, 1 d (114/6)
5000 lux, 3 d (155/10)
1000 lux, 3 d (155/12)
170
130
95
72
55
43
34
26
GAPDH
1.2
untreated (154/1)
5000 lux, 1 d (114/1)
1000 lux, 1 d (119/2)
5000 lux, 3 d (155/1)
1000 lux, 3 d (155/3)
5000 lux, 1 d (114/2)
1000 lux, 1 d (119/1)
5000 lux, 3 d (155/2)
1000 lux, 3 d (155/4)
untreated (154/4)
5000 lux, 1 d (114/3)
1000 lux, 1 d (119/4)
5000 lux, 3 d (155/5)
1000 lux, 3 d (155/7)
170
130
95
72
55
43
34
26
C1s
GAPDH
2.2
untreated (154/6)
5000 lux, 1 d (114/4)
1000 lux, 1 d (119/3)
5000 lux, 3 d (155/6)
1000 lux, 3d (155/8)
5000 lux, 1 d (114/5)
1000 lux, 1 d (119/5)
5000 lux, 3 d (155/9)
1000 lux, 3 d (155/11)
1000 lux, 1 d (119/6)
untreated (154/5)
5000 lux, 1 d (114/6)
5000 lux, 3 d (155/10)
1000 lux, 3 d (155/12)
170
130
95
72
55
43
34
26
C1s
GAPDH

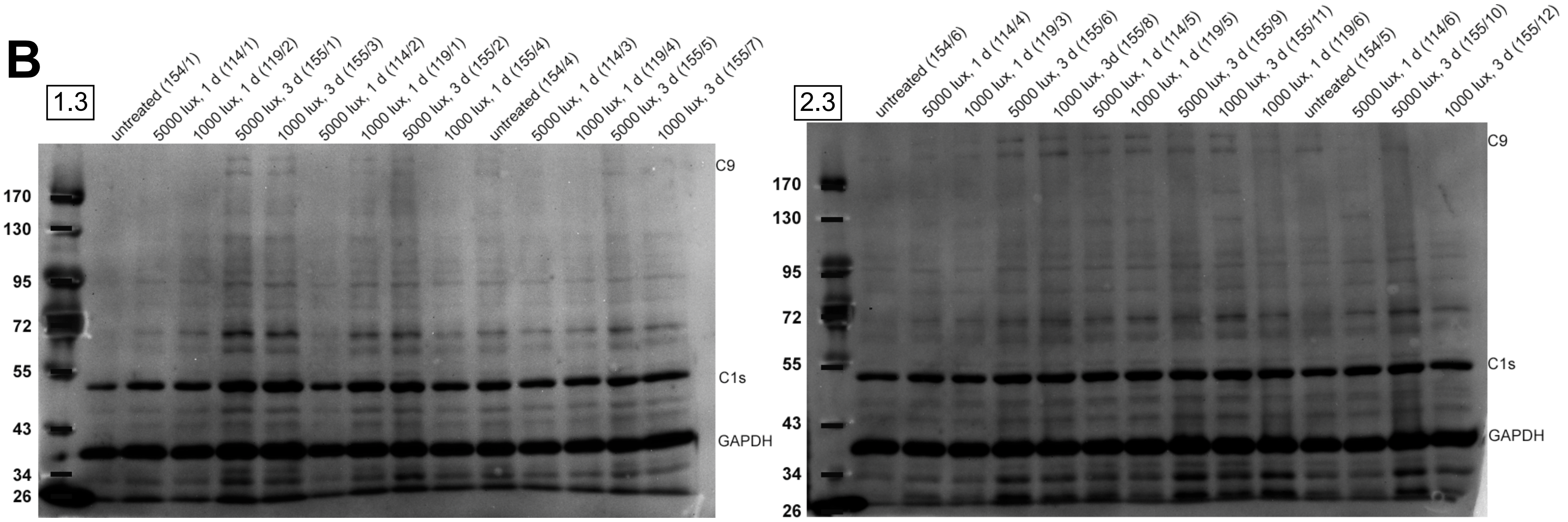
B
1.3
untreated (154/1)
5000 lux, 1 d (114/1)
1000 lux, 1 d (119/2)
5000 lux, 3 d (155/1)
1000 lux, 3 d (155/3)
5000 lux, 1 d (114/2)
1000 lux, 1 d (119/1)
5000 lux, 3 d (155/2)
1000 lux, 1 d (155/4)
untreated (154/4)
5000 lux, 1 d (114/3)
1000 lux, 1 d (119/4)
5000 lux, 3 d (155/5)
1000 lux, 3 d (155/7)
170
130
95
72
55
43
34
26
C9
C1s
GAPDH
2.3
untreated (154/6)
5000 lux, 1 d (114/4)
1000 lux, 1 d (119/3)
5000 lux, 3 d (155/6)
1000 lux, 3d (155/8)
5000 lux, 1 d (114/5)
1000 lux, 1 d (119/5)
5000 lux, 3 d (155/9)
1000 lux, 3 d (155/11)
1000 lux, 1 d (119/6)
untreated (154/5)
5000 lux, 1 d (114/6)
5000 lux, 3 d (155/10)
1000 lux, 3 d (155/12)
170
130
95
72
55
43
34
26
C9
C1s
GAPDH

C

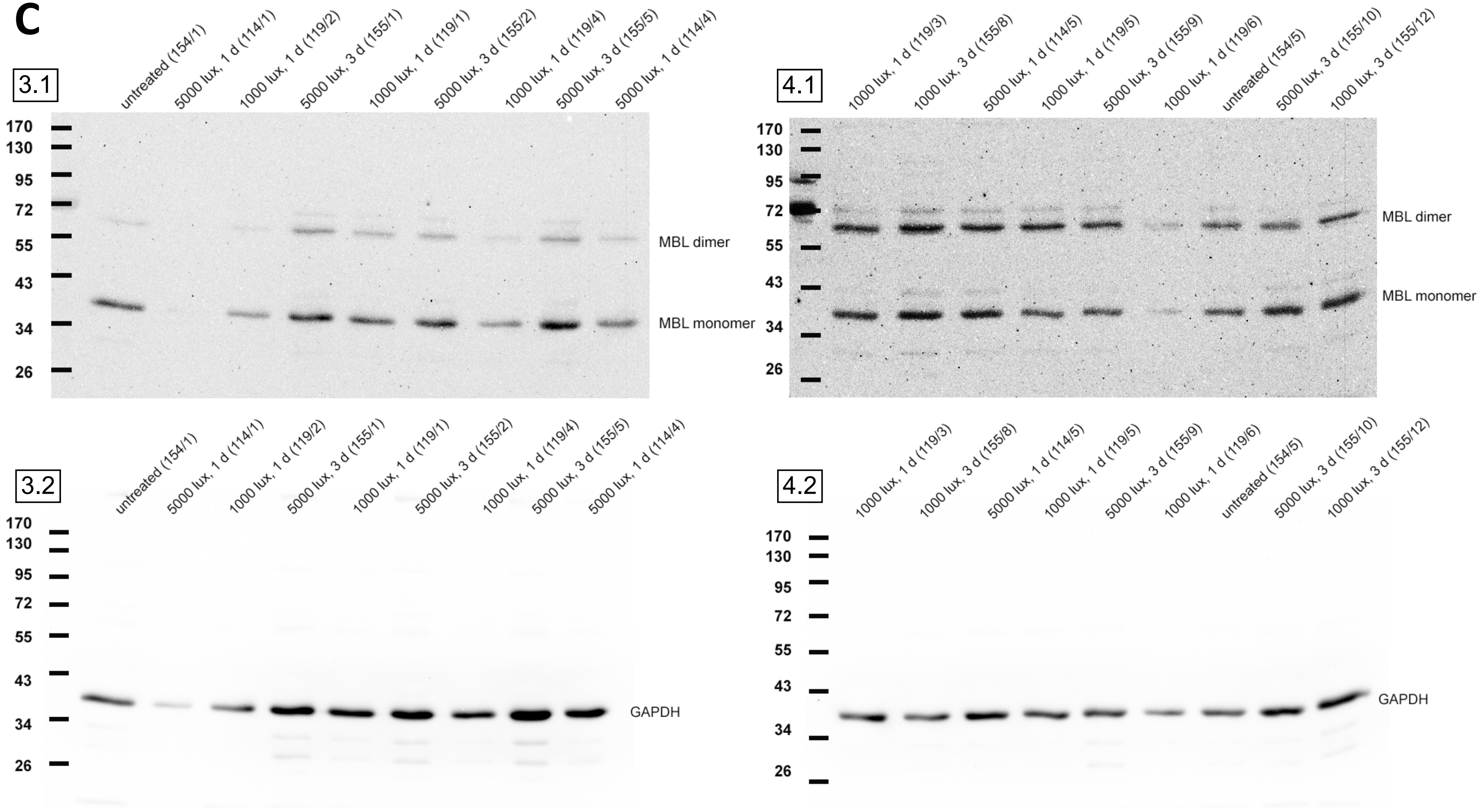
3.1
untreated (154/1)
5000 lux, 1 d (114/1)
1000 lux, 1 d (119/2)
5000 lux, 3 d (155/1)
1000 lux, 1 d (119/1)
5000 lux, 3 d (155/2)
1000 lux, 1 d (119/4)
5000 lux, 3 d (155/5)
5000 lux, 1 d (114/4)
170
130
95
72
55
43
34
26
MBL dimer
MBL monomer
4.1
1000 lux, 1 d (119/3)
1000 lux, 3 d (155/8)
5000 lux, 1 d (114/5)
1000 lux, 1 d (119/5)
5000 lux, 3 d (155/9)
1000 lux, 1 d (119/6)
untreated (154/5)
5000 lux, 3 d (155/10)
1000 lux, 3 d (155/12)
MBL dimer
MBL monomer
3.2
GAPDH
4.2
GAPDH

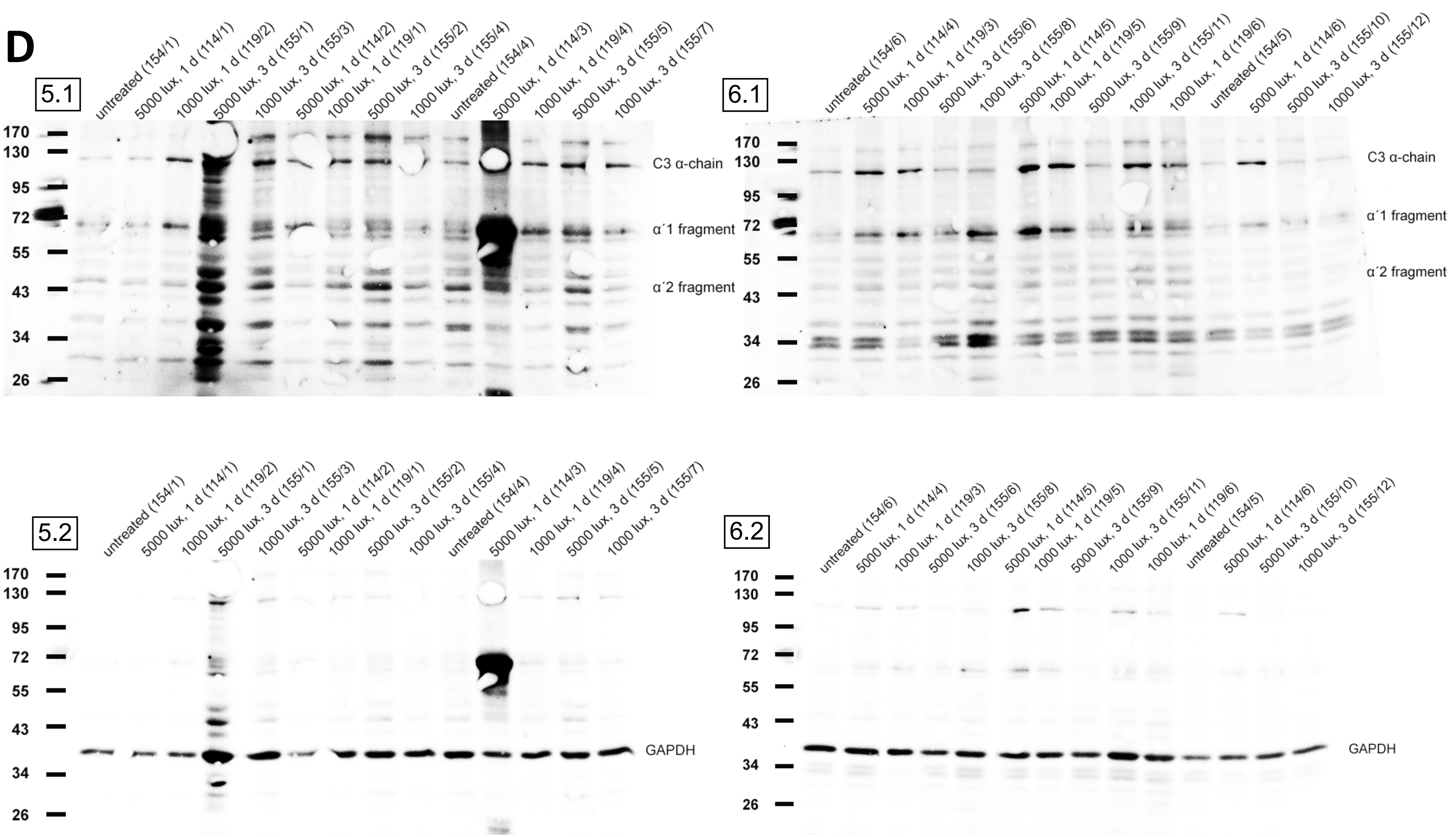
D
5.1
untreated (154/1)
5000 lux, 1 d (114/1)
1000 lux, 1 d (119/2)
5000 lux, 3 d (155/1)
1000 lux, 3 d (155/3)
5000 lux, 1 d (114/2)
1000 lux, 1 d (119/1)
5000 lux, 3 d (155/2)
1000 lux, 3 d (155/4)
untreated (154/4)
5000 lux, 1 d (114/3)
1000 lux, 1 d (119/4)
5000 lux, 3 d (155/5)
1000 lux, 3 d (155/7)
170
130
95
72
55
43
34
26
C3 α-chain
α´1 fragment
α´2 fragment
6.1
untreated (154/6)
5000 lux, 1 d (114/4)
1000 lux, 1 d (119/3)
5000 lux, 3 d (155/6)
1000 lux, 3 d (155/8)
5000 lux, 1 d (114/5)
1000 lux, 1 d (119/5)
5000 lux, 3 d (155/9)
1000 lux, 3 d (155/11)
1000 lux, 1 d (119/6)
untreated (154/5)
5000 lux, 1 d (114/6)
5000 lux, 3 d (155/10)
1000 lux, 3 d (155/12)
170
130
95
72
55
43
34
26
C3 α-chain
α´1 fragment
α´2 fragment
5.2
untreated (154/1)
5000 lux, 1 d (114/1)
1000 lux, 1 d (119/2)
5000 lux, 3 d (155/1)
1000 lux, 3 d (155/3)
5000 lux, 1 d (114/2)
1000 lux, 1 d (119/1)
5000 lux, 3 d (155/2)
1000 lux, 3 d (155/4)
untreated (154/4)
5000 lux, 1 d (114/3)
1000 lux, 1 d (119/4)
5000 lux, 3 d (155/5)
1000 lux, 3 d (155/7)
170
130
95
72
55
43
34
26
GAPDH
6.2
untreated (154/6)
5000 lux, 1 d (114/4)
1000 lux, 1 d (119/3)
5000 lux, 3 d (155/6)
1000 lux, 3 d (155/8)
5000 lux, 1 d (114/5)
1000 lux, 1 d (119/5)
5000 lux, 3 d (155/9)
1000 lux, 3 d (155/11)
1000 lux, 1 d (119/6)
untreated (154/5)
5000 lux, 1 d (114/6)
5000 lux, 3 d (155/10)
1000 lux, 3 d (155/12)
170
130
95
72
55
43
34
26
GAPDH

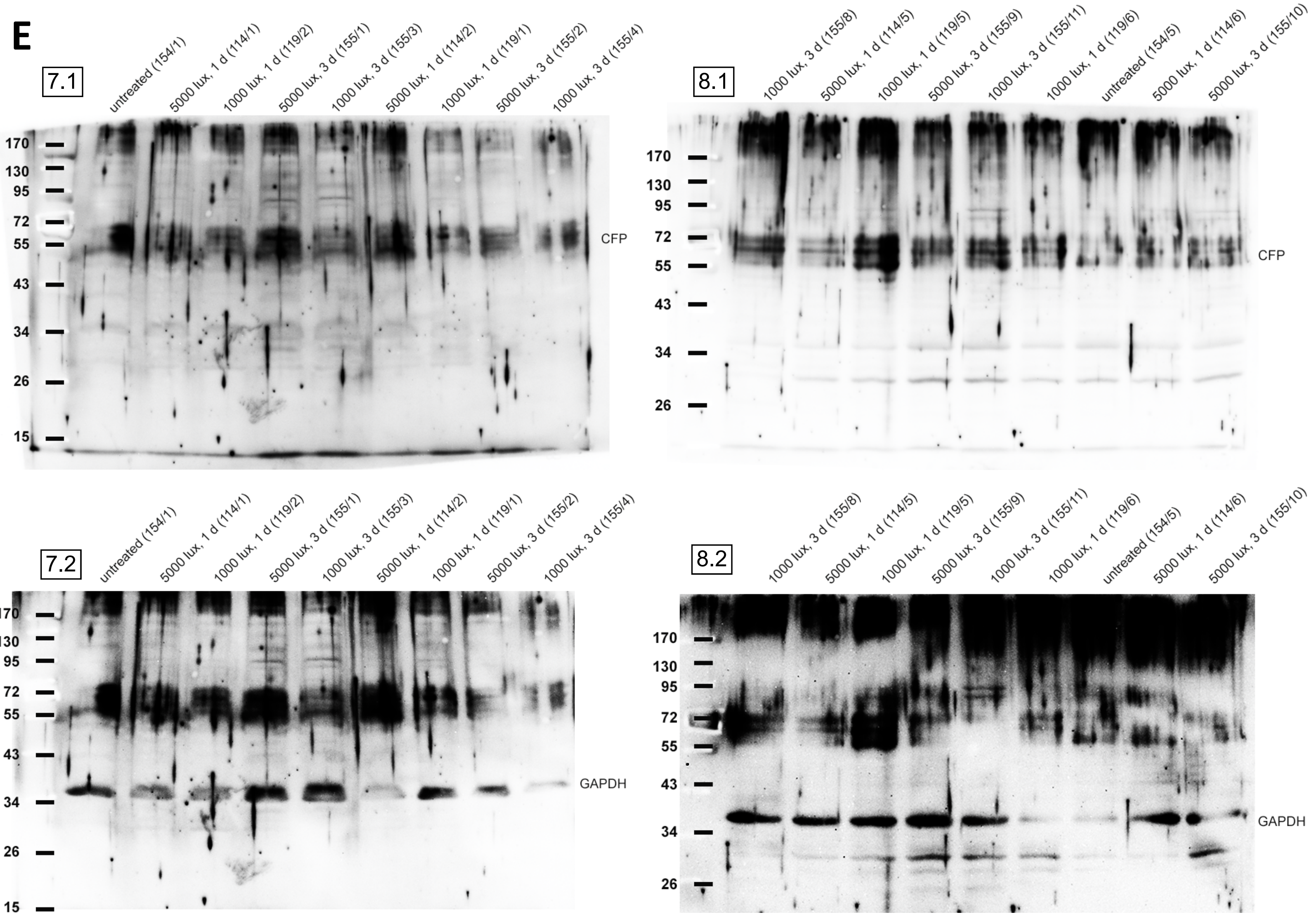
E
7.1
untreated (154/1)
5000 lux, 1 d (114/1)
1000 lux, 1 d (119/2)
5000 lux, 3 d (155/1)
1000 lux, 3 d (155/3)
5000 lux, 1 d (114/2)
1000 lux, 1 d (119/1)
5000 lux, 3 d (155/2)
1000 lux, 3 d (155/4)
170
130
95
72
55
43
34
26
15
CFP
8.1
1000 lux, 3 d (155/8)
5000 lux, 1 d (114/5)
1000 lux, 1 d (119/5)
5000 lux, 3 d (155/9)
1000 lux, 3 d (155/11)
1000 lux, 1 d (119/6)
untreated (154/5)
5000 lux, 1 d (114/6)
5000 lux, 3 d (155/10)
170
130
95
72
55
43
34
26
CFP
7.2
untreated (154/1)
5000 lux, 1 d (114/1)
1000 lux, 1 d (119/2)
5000 lux, 3 d (155/1)
1000 lux, 3 d (155/3)
5000 lux, 1 d (114/2)
1000 lux, 1 d (119/1)
5000 lux, 3 d (155/2)
1000 lux, 3 d (155/4)
170
130
95
72
55
43
34
26
15
GAPDH
8.2
1000 lux, 3 d (155/8)
5000 lux, 1 d (114/5)
1000 lux, 1 d (119/5)
5000 lux, 3 d (155/9)
1000 lux, 3 d (155/11)
1000 lux, 1 d (119/6)
untreated (154/5)
5000 lux, 1 d (114/6)
5000 lux, 3 d (155/10)
170
130
95
72
55
43
34
26
GAPDH

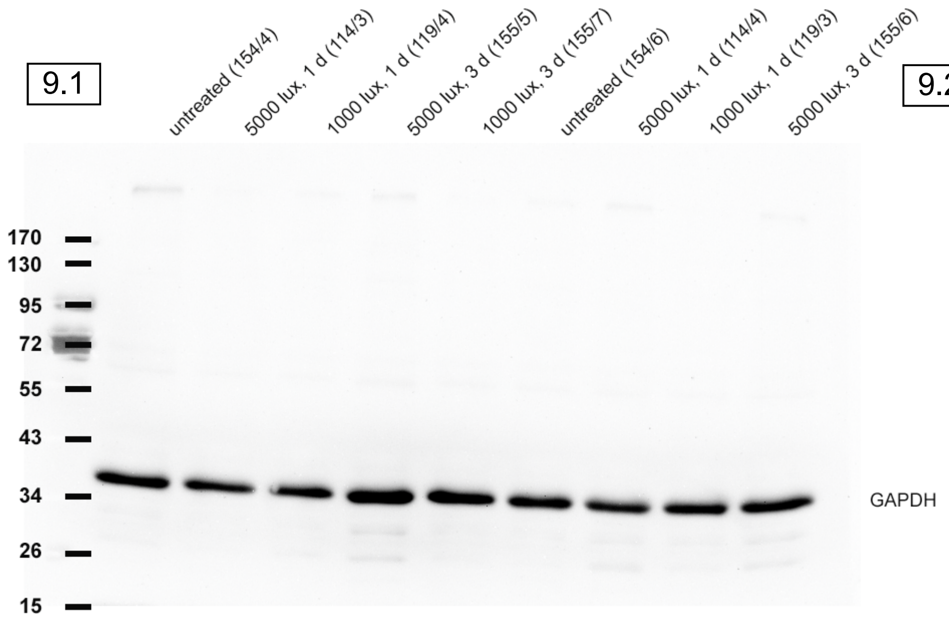

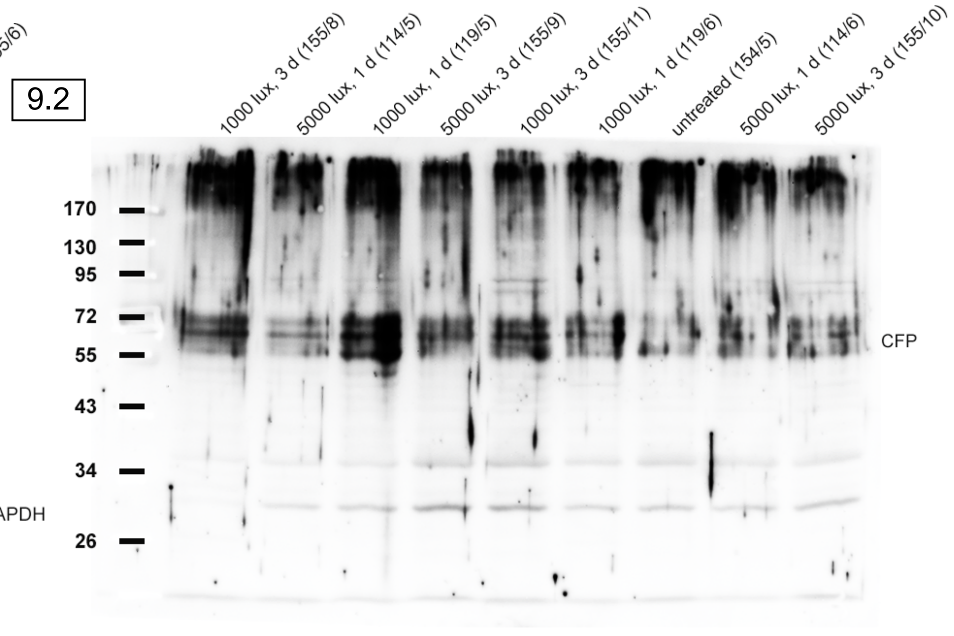

Supp. Figure 6 Entire Western Blots showing complement factor analysis for retinal tissues.

Separated retinal tissue of differently treated mice was transferred onto membranes (1 - 9). **(A)** Detection of C1s. (1.1, 2.1) Firstly, GAPDH was detected. (1.2, 2.2) Secondly, C1s was analysed. **(B)** Detection of C9. (1.3, 2.3) Thirdly, C9 was stained. **(C)** Detection of MBL. (3.1, 4.1) Firstly, MBL was detected. (3.2, 4.2) Secondly, GAPDH was analysed. **(D)** Detection of C3. (5.1, 6.1) Firstly, C3 was detected. (5.2, 6.2) Secondly, GAPDH was analysed. **(E)** Detection of CFP. (7.1, 8.1, 9.2) CFP was detected. (7.2, 8.2, 9.1) GAPDH was analysed.

A

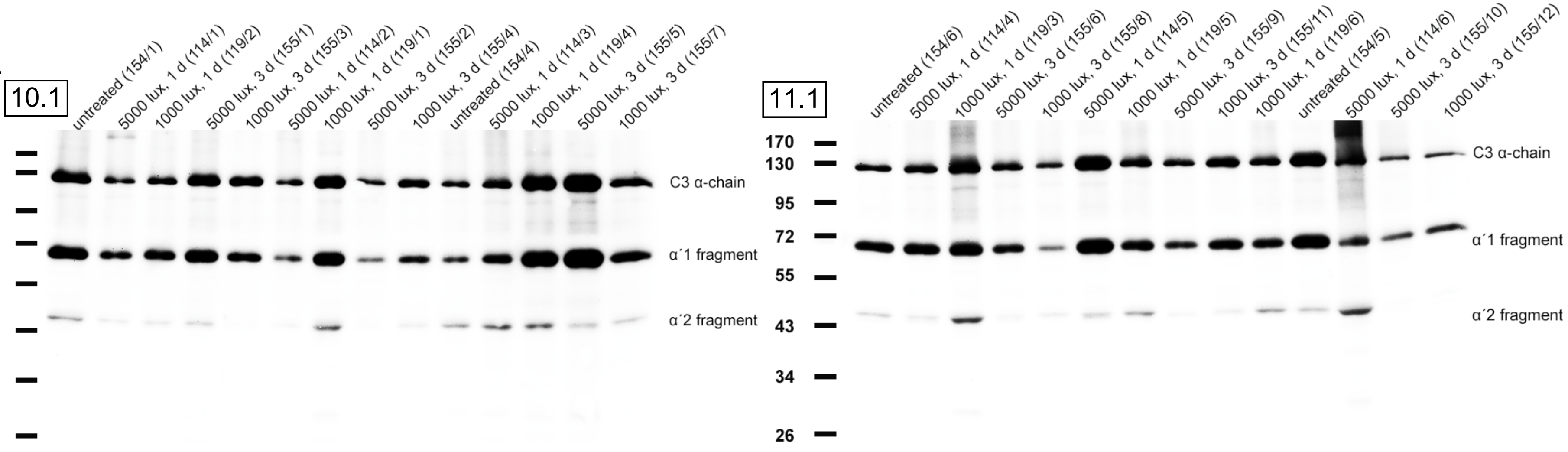
10.1
untreated (154/1)
5000 lux, 1 d (114/1)
1000 lux, 1 d (119/2)
5000 lux, 3 d (155/1)
1000 lux, 3 d (155/3)
5000 lux, 1 d (114/2)
1000 lux, 1 d (119/1)
5000 lux, 3 d (155/2)
1000 lux, 3 d (155/4)
untreated (154/4)
5000 lux, 1 d (114/3)
1000 lux, 1 d (119/4)
5000 lux, 3 d (155/5)
1000 lux, 3 d (155/7)
170
130
95
72
55
43
34
26
C3 α-chain
α´1 fragment
α´2 fragment
11.1
untreated (154/6)
5000 lux, 1 d (114/4)
1000 lux, 1 d (119/3)
5000 lux, 3 d (155/6)
1000 lux, 3 d (155/8)
5000 lux, 1 d (114/5)
1000 lux, 1 d (119/5)
5000 lux, 3 d (155/9)
1000 lux, 3 d (155/11)
1000 lux, 1 d (119/6)
untreated (154/5)
5000 lux, 1 d (114/6)
5000 lux, 3 d (155/10)
1000 lux, 3 d (155/12)
170
130
95
72
55
43
34
26
C3 α-chain
α´1 fragment
α´2 fragment

B

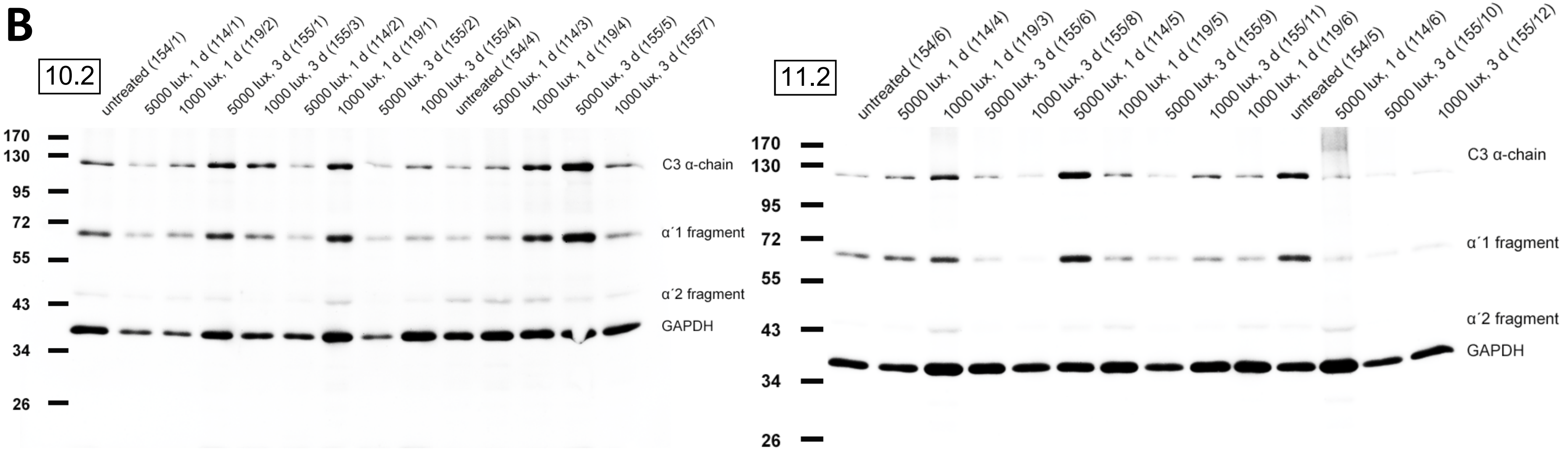
10.2
untreated (154/1)
5000 lux, 1 d (114/1)
1000 lux, 1 d (119/2)
5000 lux, 3 d (155/1)
1000 lux, 3 d (155/3)
5000 lux, 1 d (114/2)
1000 lux, 1 d (119/1)
5000 lux, 3 d (155/2)
1000 lux, 3 d (155/4)
untreated (154/4)
5000 lux, 1 d (114/3)
1000 lux, 1 d (119/4)
5000 lux, 3 d (155/5)
1000 lux, 3 d (155/7)
170
130
95
72
55
43
34
26
C3 α-chain
α´1 fragment
α´2 fragment
GAPDH
11.2
untreated (154/6)
5000 lux, 1 d (114/4)
1000 lux, 1 d (119/3)
5000 lux, 3 d (155/6)
1000 lux, 3 d (155/8)
5000 lux, 1 d (114/5)
1000 lux, 1 d (119/5)
5000 lux, 3 d (155/9)
1000 lux, 3 d (155/11)
1000 lux, 1 d (119/6)
untreated (154/5)
5000 lux, 1 d (114/6)
5000 lux, 3 d (155/10)
1000 lux, 3 d (155/12)
170
130
95
72
55
43
34
26
C3 α-chain
α´1 fragment
α´2 fragment
GAPDH

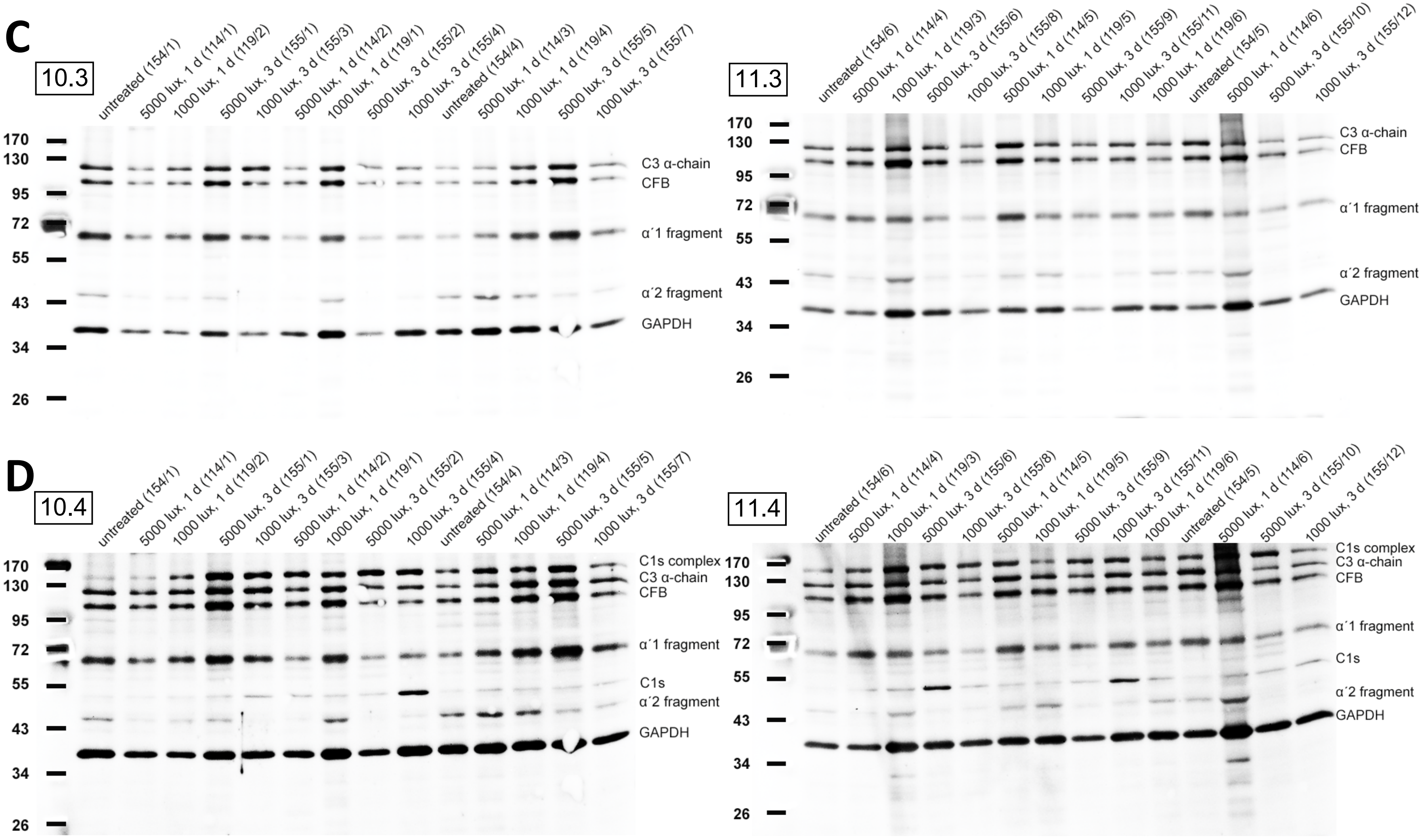
C
10.3
untreated (154/1)
5000 lux, 1 d (114/1)
1000 lux, 1 d (119/2)
5000 lux, 3 d (155/1)
1000 lux, 3 d (155/3)
5000 lux, 1 d (114/2)
1000 lux, 1 d (119/1)
5000 lux, 3 d (155/2)
1000 lux, 3 d (155/4)
untreated (154/4)
5000 lux, 1 d (114/3)
1000 lux, 1 d (119/4)
5000 lux, 3 d (155/5)
1000 lux, 3 d (155/7)
170
130
95
72
55
43
34
26
C3 α-chain
CFB
α´1 fragment
α´2 fragment
GAPDH
11.3
untreated (154/6)
5000 lux, 1 d (114/4)
1000 lux, 1 d (119/3)
5000 lux, 3 d (155/6)
1000 lux, 3 d (155/8)
5000 lux, 1 d (114/5)
1000 lux, 1 d (119/5)
5000 lux, 3 d (155/9)
1000 lux, 3 d (155/11)
1000 lux, 1 d (119/6)
untreated (154/5)
5000 lux, 1 d (114/6)
5000 lux, 3 d (155/10)
1000 lux, 3 d (155/12)
170
130
95
72
55
43
34
26
C3 α-chain
CFB
α´1 fragment
α´2 fragment
GAPDH
D
10.4
untreated (154/1)
5000 lux, 1 d (114/1)
1000 lux, 1 d (119/2)
5000 lux, 3 d (155/1)
1000 lux, 3 d (155/3)
5000 lux, 1 d (114/2)
1000 lux, 1 d (119/1)
5000 lux, 3 d (155/2)
1000 lux, 3 d (155/4)
untreated (154/4)
5000 lux, 1 d (114/3)
1000 lux, 1 d (119/4)
5000 lux, 3 d (155/5)
1000 lux, 3 d (155/7)
170
130
95
72
55
43
34
26
C1s complex
C3 α-chain
CFB
α´1 fragment
C1s
α´2 fragment
GAPDH
11.4
untreated (154/6)
5000 lux, 1 d (114/4)
1000 lux, 1 d (119/3)
5000 lux, 3 d (155/6)
1000 lux, 3 d (155/8)
5000 lux, 1 d (114/5)
1000 lux, 1 d (119/5)
5000 lux, 3 d (155/9)
1000 lux, 3 d (155/11)
1000 lux, 1 d (119/6)
untreated (154/5)
5000 lux, 1 d (114/6)
5000 lux, 3 d (155/10)
1000 lux, 3 d (155/12)
170
130
95
72
55
43
34
26
C1s complex
C3 α-chain
CFB
α´1 fragment
C1s
α´2 fragment
GAPDH

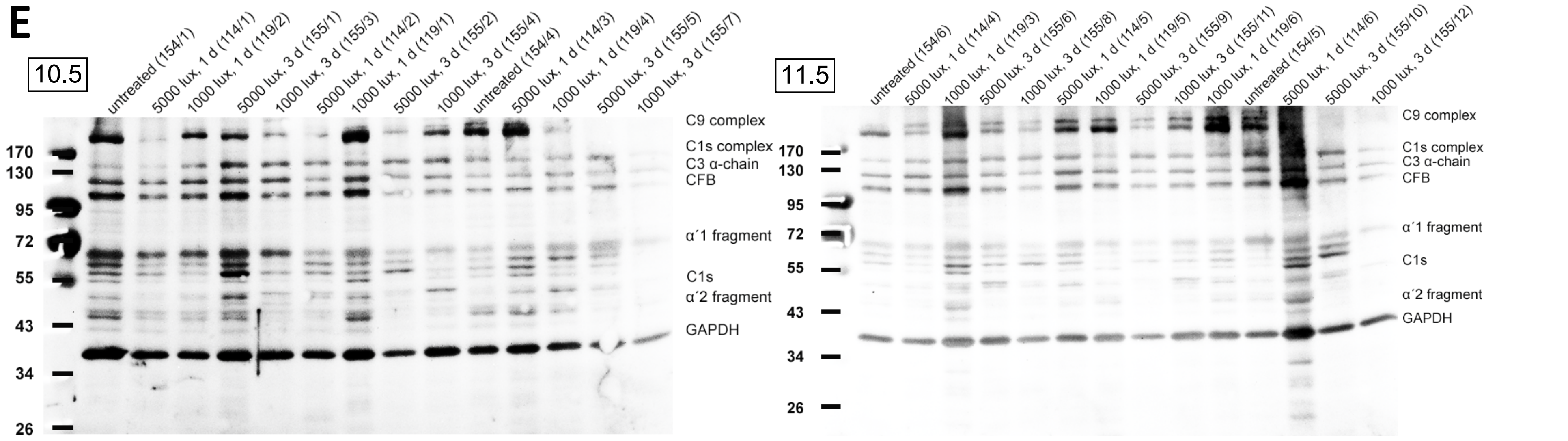
E
10.5
untreated (154/1)
5000 lux, 1 d (114/1)
1000 lux, 1 d (119/2)
5000 lux, 3 d (155/1)
1000 lux, 3 d (155/3)
5000 lux, 1 d (114/2)
1000 lux, 1 d (119/1)
5000 lux, 3 d (155/2)
1000 lux, 3 d (155/4)
untreated (154/4)
5000 lux, 1 d (114/3)
1000 lux, 1 d (119/4)
5000 lux, 3 d (155/5)
1000 lux, 3 d (155/7)
170
130
95
72
55
43
34
26
C9 complex
C1s complex
C3 α-chain
CFB
α´1 fragment
C1s
α´2 fragment
GAPDH
11.5
untreated (154/6)
5000 lux, 1 d (114/4)
1000 lux, 1 d (119/3)
5000 lux, 3 d (155/6)
1000 lux, 3 d (155/8)
5000 lux, 1 d (114/5)
1000 lux, 1 d (119/5)
5000 lux, 3 d (155/9)
1000 lux, 3 d (155/11)
1000 lux, 1 d (119/6)
untreated (154/5)
5000 lux, 1 d (114/6)
5000 lux, 3 d (155/10)
1000 lux, 3 d (155/12)
170
130
95
72
55
43
34
26
C9 complex
C1s complex
C3 α-chain
CFB
α´1 fragment
C1s
α´2 fragment
GAPDH

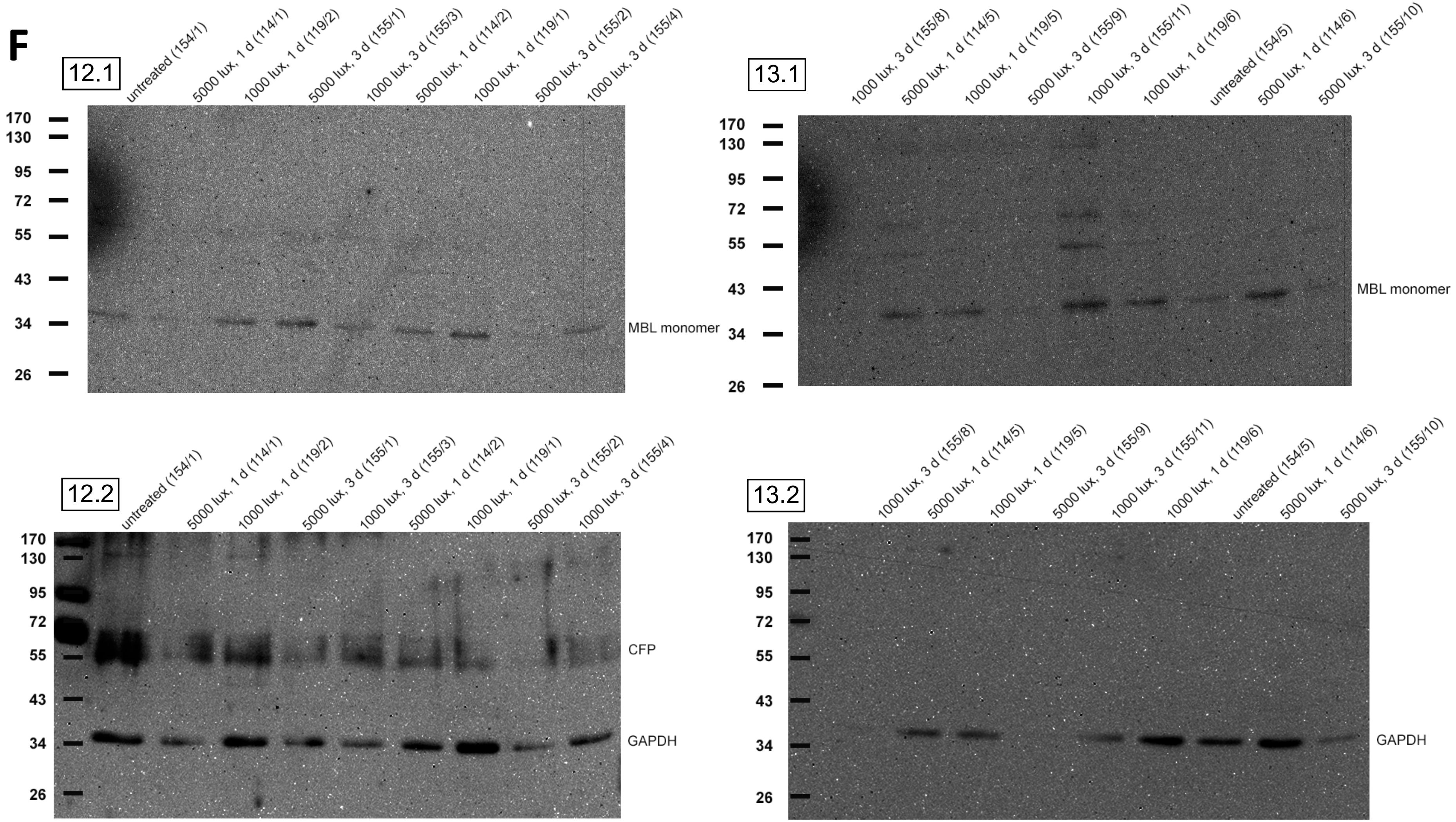
F
12.1
untreated (154/1)
5000 lux, 1 d (114/1)
1000 lux, 1 d (119/2)
5000 lux, 3 d (155/1)
1000 lux, 3 d (155/3)
5000 lux, 1 d (114/2)
1000 lux, 1 d (119/1)
5000 lux, 3 d (155/2)
1000 lux, 3 d (155/4)
170
130
95
72
55
43
34
26
MBL monomer
13.1
1000 lux, 3 d (155/8)
5000 lux, 1 d (114/5)
1000 lux, 1 d (119/5)
5000 lux, 3 d (155/9)
1000 lux, 3 d (155/11)
1000 lux, 1 d (119/6)
untreated (154/5)
5000 lux, 1 d (114/6)
5000 lux, 3 d (155/10)
170
130
95
72
55
43
34
26
MBL monomer
12.2
untreated (154/1)
5000 lux, 1 d (114/1)
1000 lux, 1 d (119/2)
5000 lux, 3 d (155/1)
1000 lux, 3 d (155/3)
5000 lux, 1 d (114/2)
1000 lux, 1 d (119/1)
5000 lux, 3 d (155/2)
1000 lux, 3 d (155/4)
170
130
95
72
55
43
34
26
CFP
GAPDH
13.2
1000 lux, 3 d (155/8)
5000 lux, 1 d (114/5)
1000 lux, 1 d (119/5)
5000 lux, 3 d (155/9)
1000 lux, 3 d (155/11)
1000 lux, 1 d (119/6)
untreated (154/5)
5000 lux, 1 d (114/6)
5000 lux, 3 d (155/10)
170
130
95
72
55
43
34
26
GAPDH

G

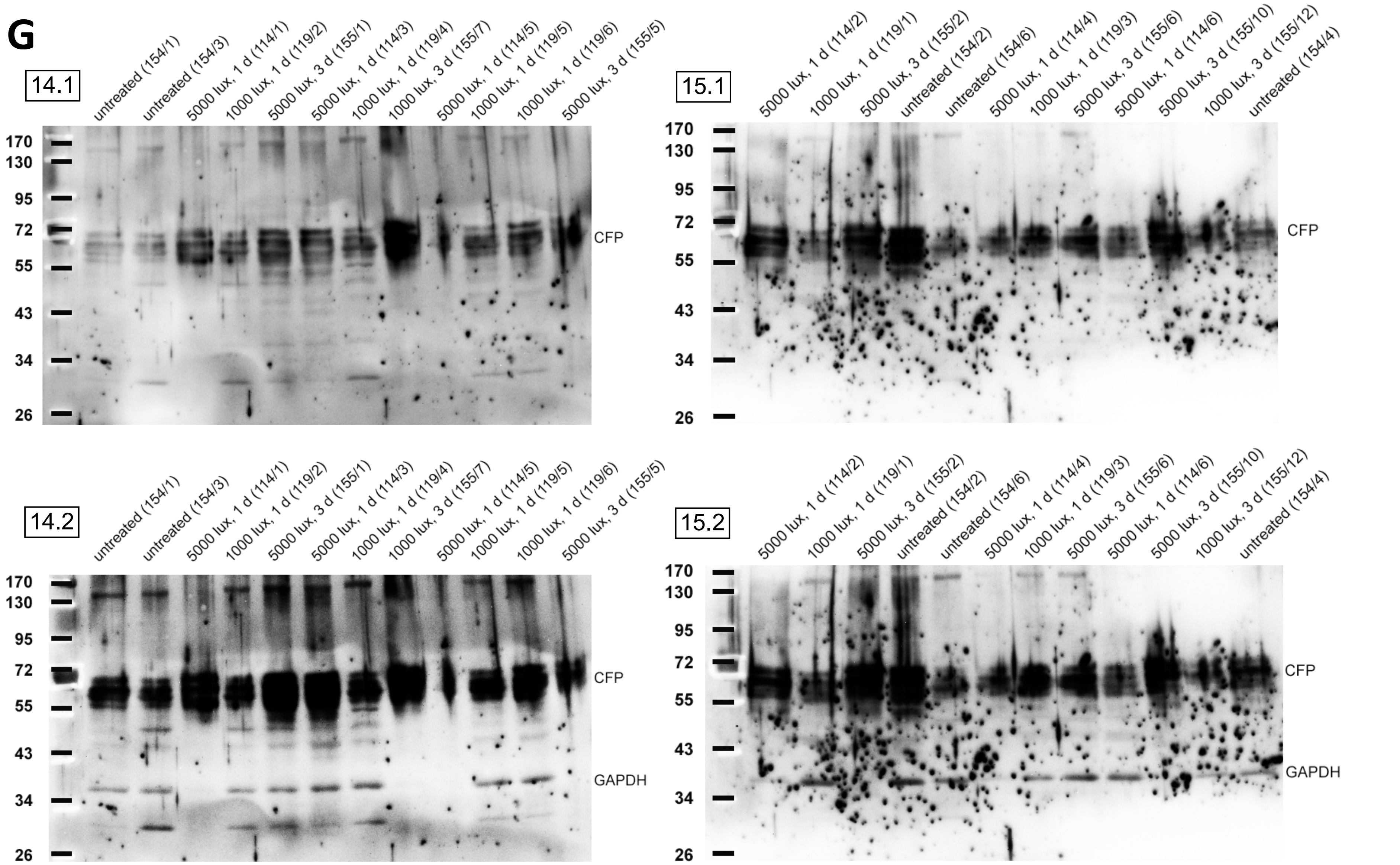
14.1
untreated (154/1)
untreated (154/3)
5000 lux, 1 d (114/1)
1000 lux, 1 d (119/2)
5000 lux, 3 d (155/1)
5000 lux, 1 d (114/3)
1000 lux, 1 d (119/4)
1000 lux, 3 d (155/7)
5000 lux, 1 d (114/5)
1000 lux, 1 d (119/5)
1000 lux, 1 d (119/6)
5000 lux, 3 d (155/5)
170
130
95
72
55
43
34
26
CFP
15.1
5000 lux, 1 d (114/2)
1000 lux, 1 d (119/1)
5000 lux, 3 d (155/2)
untreated (154/2)
untreated (154/6)
5000 lux, 1 d (114/4)
1000 lux, 1 d (119/3)
5000 lux, 3 d (155/6)
5000 lux, 1 d (114/6)
5000 lux, 3 d (155/10)
1000 lux, 3 d (155/12)
untreated (154/4)
170
130
95
72
55
43
34
26
CFP
14.2
untreated (154/1)
untreated (154/3)
5000 lux, 1 d (114/1)
1000 lux, 1 d (119/2)
5000 lux, 3 d (155/1)
5000 lux, 1 d (114/3)
1000 lux, 1 d (119/4)
1000 lux, 3 d (155/7)
5000 lux, 1 d (114/5)
1000 lux, 1 d (119/5)
1000 lux, 1 d (119/6)
5000 lux, 3 d (155/5)
170
130
95
72
55
43
34
26
CFP
GAPDH
15.2
5000 lux, 1 d (114/2)
1000 lux, 1 d (119/1)
5000 lux, 3 d (155/2)
untreated (154/2)
untreated (154/6)
5000 lux, 1 d (114/4)
1000 lux, 1 d (119/3)
5000 lux, 3 d (155/6)
5000 lux, 1 d (114/6)
5000 lux, 3 d (155/10)
1000 lux, 3 d (155/12)
untreated (154/4)
170
130
95
72
55
43
34
26
CFP
GAPDH

H

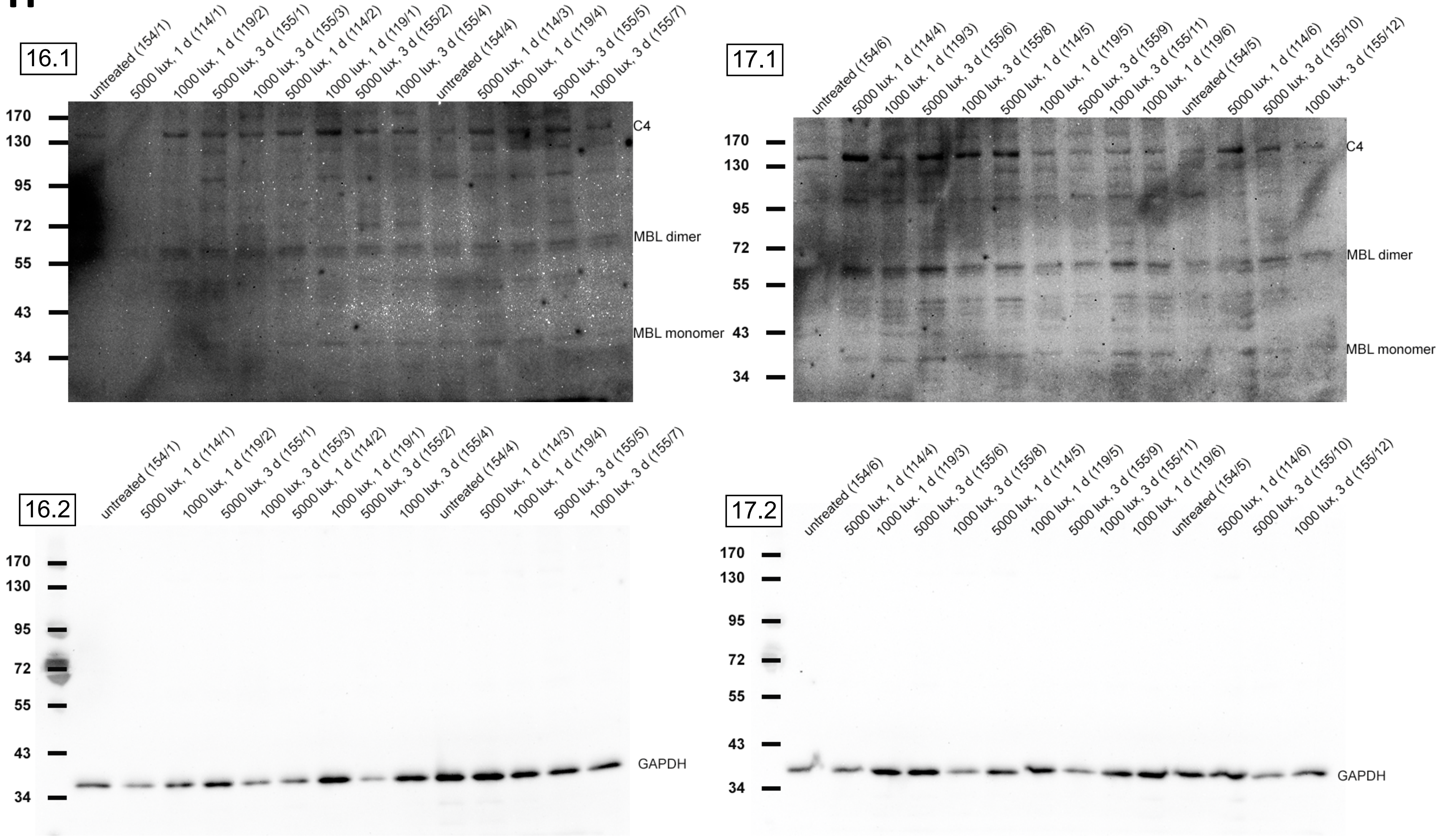
16.1
untreated (154/1)
5000 lux, 1 d (114/1)
1000 lux, 1 d (119/2)
5000 lux, 3 d (155/1)
1000 lux, 3 d (155/3)
5000 lux, 1 d (114/2)
1000 lux, 1 d (119/1)
5000 lux, 3 d (155/2)
1000 lux, 3 d (155/4)
untreated (154/4)
5000 lux, 1 d (114/3)
1000 lux, 1 d (119/4)
5000 lux, 3 d (155/5)
1000 lux, 3 d (155/7)
170
130
95
72
55
43
34
C4
MBL dimer
MBL monomer
17.1
untreated (154/6)
5000 lux, 1 d (114/4)
1000 lux. 1 d (119/3)
5000 lux, 3 d (155/6)
1000 lux, 3 d (155/8)
5000 lux, 1 d (114/5)
1000 lux, 1 d (119/5)
5000 lux, 3 d (155/9)
1000 lux, 3 d (155/11)
1000 lux, 1 d (119/6)
untreated (154/5)
5000 lux, 1 d (114/6)
5000 lux, 3 d (155/10)
1000 lux, 3 d (155/12)
170
130
95
72
55
43
34
C4
MBL dimer
MBL monomer
16.2
untreated (154/1)
5000 lux, 1 d (114/1)
1000 lux, 1 d (119/2)
5000 lux, 3 d (155/1)
1000 lux, 3 d (155/3)
5000 lux, 1 d (114/2)
1000 lux, 1 d (119/1)
5000 lux, 3 d (155/2)
1000 lux, 3 d (155/4)
untreated (154/4)
5000 lux, 1 d (114/3)
1000 lux, 1 d (119/4)
5000 lux, 3 d (155/5)
1000 lux, 3 d (155/7)
170
130
95
72
55
43
34
GAPDH
17.2
untreated (154/6)
5000 lux, 1 d (114/4)
1000 lux. 1 d (119/3)
5000 lux, 3 d (155/6)
1000 lux, 3 d (155/8)
5000 lux, 1 d (114/5)
1000 lux, 1 d (119/5)
5000 lux, 3 d (155/9)
1000 lux, 3 d (155/11)
1000 lux, 1 d (119/6)
untreated (154/5)
5000 lux, 1 d (114/6)
5000 lux, 3 d (155/10)
1000 lux, 3 d (155/12)
170
130
95
72
55
43
34
GAPDH

Supp. Figure 7 Entire Western Blots showing complement factor analysis for RPE/choroid tissues.

RPE/choroid tissues of differently treated mice were transferred onto membranes (10 - 17). **(A)** Detection of C3. (10.1, 11.1) Firstly, C3 was detected. **(B)** (10.2, 11.2) Secondly, GAPDH was analysed. **(C)** (10.3, 11.3) Thirdly, CFB was stained. **(D)** (10.4, 11.4) Fourthly, C1s was detected. **(E)** (10.5, 11.5) Fifthly, C9 was analysed. **(F)** Detection of MBL. (12.1, 13.1) Firstly, MBL was detected. (12.2) Secondly, GAPDH and CFP were stained. (13.2) GAPDH was analysed. **(G)** Detection of CFP. (14.1, 15.1) Firstly, CFP was detected. (14.2, 15.2) Secondly, GAPDH was analysed. **(H)** Detection of C4. (16.1, 17.1) Firstly, MBL was stained. Secondly, C4 was detected. (16.2, 17.2) Thirdly, GAPDH was analysed.

Supp. Table S1 Specific anti-mouse primary antibodies and species-specific secondary antibodies

primary antibody	species	company	catalogue number / Reference	dilution/ conc.
anti-C1s	rabbit	Proteintech (Rosemont, IL, USA)	#14554-1-AP	1 : 100
anti-C3-HRP	goat	MP Biomedicals (Santa Ana, CA, USA)	#55557	1 : 1000
anti-C3a	rat	BD Biosciences (Heidelberg, Germany)	#558250	3 µg/mL
anti-C3a-biotin	rat	BD Biosciences (Heidelberg, Germany)	#558251	3 µg/mL
anti-C3d	goat	R&D systems (Wiesbaden, Germany)	#AF2655/ [1][2]	1 : 75
anti-C4	rat	Abcam (Cambridge, UK)	#ab11863	1 : 50
anti-C5a	rat	BD Biosciences (Heidelberg, Germany)	#558027	2 µg/mL
anti-C5a-biotin	rat	BD Biosciences (Heidelberg, Germany)	#558028	2 µg/mL
anti-C9	rabbit	Antikoerper-online (Aachen, Germany)	#ABIN1714714	1 : 200
anti-C5b-C9	rabbit	Abcam (Cambridge, UK)	#ab55811/ [3]	1: 50
anti-CFB	goat	Calbiochem/Merck Millipore (Darmstadt, Germany)	#341272	1 : 5000
anti-MBL-A	rabbit	Abcam (Cambridge, UK)	#ab133629	1 : 1000
anti-mCFP	rat	in house	-	1 : 50
anti-GAPDH-HRP	rabbit	Cell Signaling Technology (Danvers, MA, USA)	#3683	1 : 1000
anti-Iba-1	rabbit	Wako Chemicals (Neuss, Germany)	#019-19741	1 : 600
anti-GFAP	rabbit	Abcam (Cambridge, UK)	#ab7260	1 : 300
**secondary antibody**				
anti-rabbit IgG-HRP	goat	Dianova (Hamburg, Germany)	#111-035-003	1 : 10000

anti-rabbit-Polymer-AP	unknown	Zytomed (Berlin, Germany)	#ZUC031-006	-
anti-rabbit-488	goat	Thermo Fisher Scientific (Braunschweig, Germany)	#A-11008	1 : 1000
anti-rat IgG-HRP	goat	Dianova (Hamburg, Germany)	#112-035-003	1 : 5000
anti-goat IgG-HRP	rabbit	Dianova (Hamburg, Germany)	#305-035-003	1 : 5000
anti-goat-A546	rabbit	Thermo Fisher Scientific (Braunschweig, Germany)	#A-21085	1 : 1000

Supp. Table S2 Oligonucleotide primer pairs for qRT-PCR analysis and amplification product size for RT-PCR analysis

transcript	primer sequence (5' – 3')	product size (bp)
c1s	Forward: CCCTGTAGCCACTTCTGCAA<br>Reverse: GGGCAGTGAACACATCTCCA	124
c3	Forward: AGCCCAACACCAGCTACATC<br>Reverse: GAATGCCCCAAGTTCTTCGC	113
c4	Forward: TCTGAAGCCTCCAACGTTCC<br>Reverse: TGGGATGGGGAAGGAAATGC	126
c9	Forward: TGGTGACAACGACTGTGGAG<br>Reverse: TGTTGATCCCATAGCCTGCTG	125
cfb	Forward: GGTGCCTCACCAACTTGATT<br>Reverse: CTTGGTGTTGGTCCCTGACT	197
mbl-a	Forward: TTCCCGGTCACCAGGCTAAT<br>Reverse: GAAGCATGGTCCTTACTAGGGT	123
masp-1	Forward: TGGGGGTAGCCTTTTAGGTTC<br>Reverse: AGGGCTGAGCAAGTATGA	109
cfp	Forward: AGGTGCAAAGGCCTACTTGG<br>Reverse: TGACCATTGTGGAGACCTGC	120
actin	Forward: ATGCTCCCCGGGCTGTAT<br>Reverse: CATAGGAGTCCTTCTGACCCATTC	87
lif1	Forward: AAACGGCCTGCATCTAAGG<br>Reverse: AGCAGCAGTAAGGGCACAAT	93
gfap	Forward: TCGAGATCGCCACCTACAG<br>Reverse: GTCTGTACAGGAATGGTGATGC	102

References Supplement

1. Li Y, Song D, Song Y, Zhao L, Wolkow N, Tobias JW, et al. Iron-induced local complement component 3 (C3) up-regulation via non-canonical transforming growth factor (TGF)-β signaling in the retinal pigment epithelium. J. Biol. Chem. 2015;290:11918–34.

2. Leung VWY, Yun S, Botto M, Mason JC, Malik TH, Song W, et al. Decay-accelerating factor suppresses complement C3 activation and retards atherosclerosis in low-density lipoprotein receptor-deficient mice. Am. J. Pathol. 2009;175:1757–67.

3. Ratajczak MZ, Lee H, Wysoczynski M, Wan W, Marlicz W, Laughlin MJ, et al. Novel insight into stem cell mobilization-plasma sphingosine-1-phosphate is a major chemoattractant that directs the egress of hematopoietic stem progenitor cells from the bone marrow and its level in peripheral blood increases during mobilization due to. Leukemia. 2010;24:976–85.
